# Singular and short-term anesthesia exposure in the developing brain induces persistent neuronal changes consistent with chronic neurodegenerative disease

**DOI:** 10.1038/s41598-021-85125-5

**Published:** 2021-03-11

**Authors:** Kaley Hogarth, Ramesh Babu Vanama, Greg Stratmann, Jason T. Maynes

**Affiliations:** 1 Division of Molecular Medicine, SickKids Research Institute, Toronto, Canada; 2 Department of Anesthesia and Pain Medicine, Hospital for Sick Children, 555 University Ave., Toronto, ON M5G 1X8 Canada; 3 Department of Anesthesia and Perioperative Medicine, University of California, San Francisco, San Francisco, USA; 4 Department of Anesthesiology and Pain Medicine, University of Toronto, Toronto, Canada

**Keywords:** Experimental models of disease, Molecular medicine, Biochemistry, Mitochondrial proteins, Protein folding, Mechanisms of disease, Energy metabolism

## Abstract

The potential adverse impact of inhalational anesthetics on the developing brain was highlighted by the addition of a medication warning by the U.S. Food and Drug Administration for their use in the pediatric population. To investigate mechanisms by which early life anesthesia exposure could induce long-term neuronal dysfunction, we exposed rats to 1 minimum alveolar concentration sevoflurane at 7 days of life. The animals were raised normally until adulthood (P300) prior to sacrifice and analysis of cortical tissue structure (TEM), mitochondrial quality control and biogenesis pathways (Western blot, ELISA, ADP/ATP content), and markers of oxidative stress, proteotoxicity and inflammation (Western blot, ELISA). We found that early life anesthesia exposure led to adverse changes in mitochondrial quality maintenance pathways, autophagy and mitochondrial biogenesis. Although there was an escalation of oxidative stress markers and an increase in the nuclear localization of stress-related transcription factors, cellular redox compensatory responses were blunted, and oxidative phosphorylation was reduced. We found upregulation of mitochondrial stress and proteotoxicity markers, but a significant reduction of mitochondrial unfolded protein response end-effectors, contributing to an increase in inflammation. Contrary to acute exposure, we did not find an increase in apoptosis. Our findings suggest that a limited, early exposure to anesthesia may produce lasting cellular dysfunction through the induction of a sustained energy deficient state, resulting in persistent neuroinflammation and altered proteostasis/toxicity, mimicking aspects of chronic neurodegenerative diseases.

## Introduction

In 2016, the Food and Drug Administration (FDA) added a medication warning label to inhalational anesthetics, indicating a potential risk for adverse long-term neurocognitive outcomes with repeated or extended exposures in young children and in pregnant mothers (with risk to the unborn fetus)^1^. This warning is scientifically based on a combination of retrospective clinical studies, generally highlighting the risks to young patients receiving more than one anesthetic, but these studies are unable to inform on specific mechanisms since the anesthetic regimens were not controlled^2–5^. In a recent prospective, multisite trial, the effect of a single general anesthesia (GA) exposure was compared to neuraxial anesthesia^6^. The results from this trial agree with the previously described retrospective studies, in that a single, short exposure to GA in an otherwise healthy young child likely does not have an adverse effect. While investigating the potential effect of duration (or number) of anesthetics, these clinical studies do not interrogate causative pharmaceuticals or physiologic processes, in pathways that could plausibly result in the long temporal association between drug exposure and the observed cognitive phenotype.

To address this association, pre-clinical investigations have attempted to elucidate an underlying mechanism, controlling for variables which may also affect neurocognitive development and confound retrospective clinical studies. Two early studies in rat models illustrated that anesthetic agents acutely induced widespread neuronal apoptosis^7,8^, findings which were later extended to non-human primates^9^. Similarly, small animal models illustrated that early exposure to inhalational anesthetic agents could adversely affect cognition and learning^10–13^. The potential mechanisms explored include gross neuronal apoptosis^14,15^, dendritic pruning^16^, changes to neuro-/synaptogenesis^17^, mitochondrial dysfunction and the generation of reactive oxygen species (ROS)^18^, alteration to brain derived neurotrophic factor/p75^NTR^ signalling^19^, and AMPA receptor modulation^20^, among others. However, most of the observed cellular, tissue or organism changes are in very close proximity to the anesthetic administration, making the link to long-term neurocognitive function challenging to elucidate, and not allowing for the description of techniques or therapies that might mitigate the adverse effects.

To address this knowledge gap, we utilized our previously described animal model of neurocognitive development, where sevoflurane exposure in early life (P7) was shown to affect learning and induce long-term adverse neurocognitive changes^11,13,21,22^. Through analysis of cortical tissue structure (TEM), mitochondrial quality control and biogenesis pathways (Western blot, ELISA, ADP/ATP content), and markers of oxidative stress, proteotoxicity and inflammation (Western blot, ELISA), we illustrate that a singular anesthetic exposure produces adverse changes that persist into adulthood. The observed changes include the induction of enduring neuroinflammation and mitochondrial injury, mimicking aspects of chronic neurodegenerative diseases, providing a potentially addressable mechanism for the associated temporally distant neurological dysfunction.

## Results

### Adult cortical neuron mitochondrial ultrastructure and size are altered after infant sevoflurane exposure

**Figure 1:**
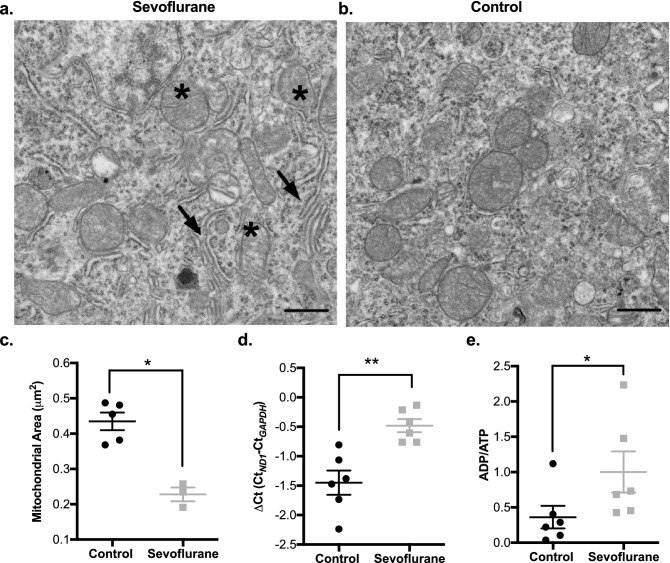
Infant (P7) sevoflurane exposure alters mitochondrial ultrastructure and energetic status in adult rat cortical tissue. Transmission electron microscopy (TEM) imaging illustrates significant changes to mitochondrial ultrastructure including swollen cristae (*), dilated rough endoplasmic reticulum (arrows) and a decreased average mitochondrial size (increase in fragmented phenotype) with infant sevoflurane exposure. (**a**) representative P7 sevoflurane-exposed adult rat cortical brain TEM image, (**b**) littermate control adult rat cortical brain TEM image. In cortical tissue, sevoflurane treatment resulted in a reduction in (**c**) average mitochondrial size (area, from TEM) and (**d**) relative cortical mitochondrial DNA content (ΔC_t_ comparing mitochondrial *ND1* to nuclear *GAPDH*). (**e**) Sevoflurane reduces energy availability, illustrated through an increased ADP/ATP. TEM magnification ×20,000, scale bars represent 500 nm. Values represent means ± SEM, with 6 animals in each group. Statistical significance between groups indicated with (*) for *p*-value < 0.05, and (**) for *p-*value < 0.01.

Mitochondria undergo the antagonistic dynamic processes of fission and fusion in order to appropriately respond to cellular metabolic needs and external stimuli, and for repair processes^23^. To examine if an early exposure to inhalational anesthetics could alter this structure–function relationship in adult neuronal mitochondria, we examined the organelle’s ultrastructure using electron microscopy (Fig. 1a,b). We found that mitochondria from adult animals with sevoflurane exposure had a significantly smaller average area (37% reduction in size, *p* = 0.01) and an altered, swollen cristae architecture (Fig. 1a–c). The fragmented mitochondrial population contrasts with the fused and dilated mitochondria noted after acute inhalational anesthetic exposure, indicating a temporal evolution of mitochondrial morphology^24^.

**Figure 2:**
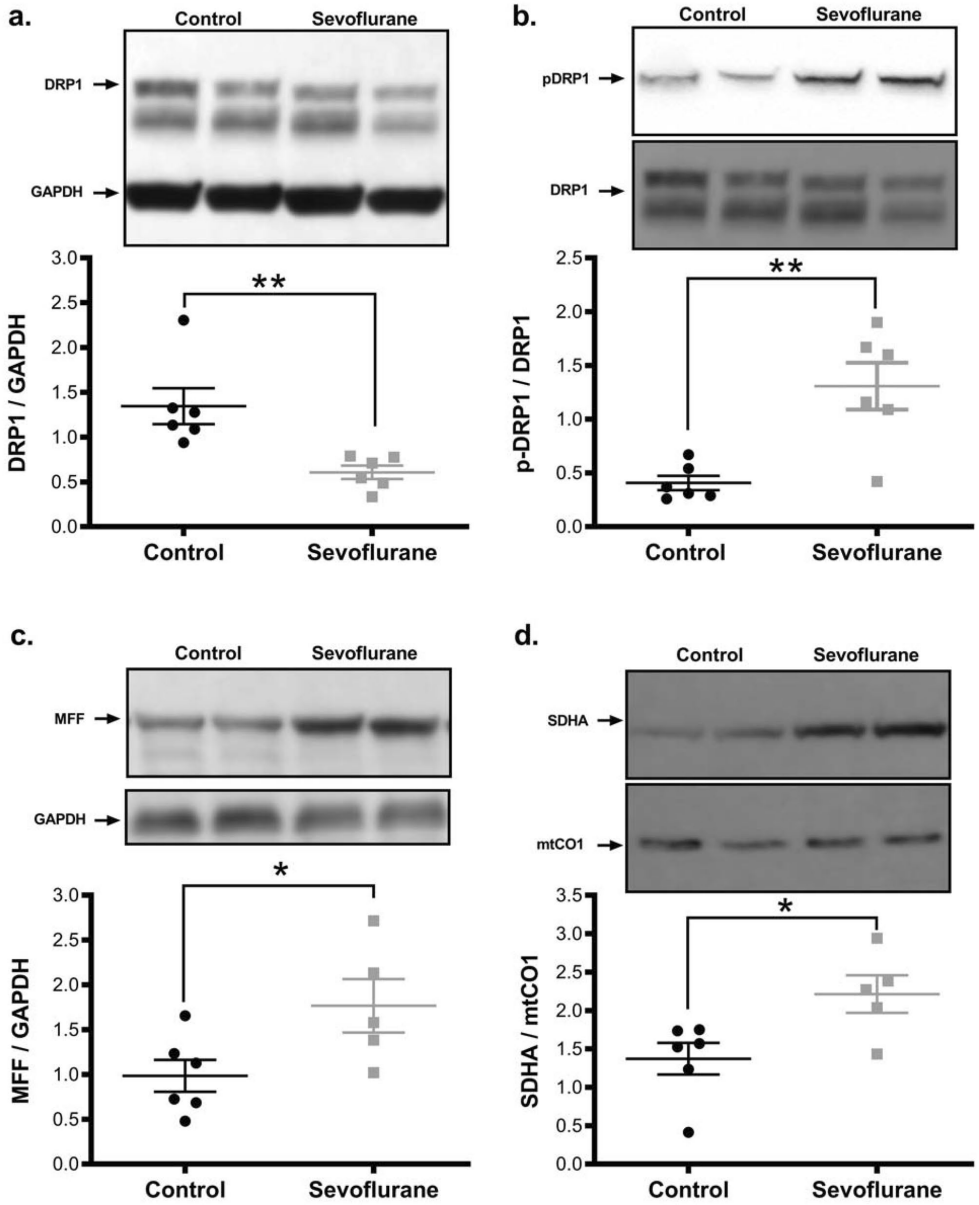
Sevoflurane exposure at P7 promotes mitochondrial fission and induces mitonuclear protein imbalance in the cortical tissue of adult rats. Sevoflurane exposure reduced the total level of the pro-fission GTPase DRP1 (**a**), but dramatically increased the level of the activated, phosphorylated form (pSer616-DRP1) (**b**). Similarly, the pro-fission DRP1 adapter MFF was elevated with sevoflurane exposure (**c**). Sevoflurane altered mitochondrial homeostasis, with an increase in nuclear-encoded protein components of the electron transport chain (Succinate Dehydrogenase Complex A-SDHA) relative to the mitochondrial-encoded components (Mitochondrial Cytochrome C Oxidase 1-mtCO1), adversely altering the mitonuclear protein balance (lower proportion of mitochondrial-encoded proteins)^27^. (**d**). Values represent means ± SEM, with 6 animals in each group. Statistical significance between groups indicated with (*) for *p*-value < 0.05, and (**) for *p-*value < 0.01.

**Figure 3:**
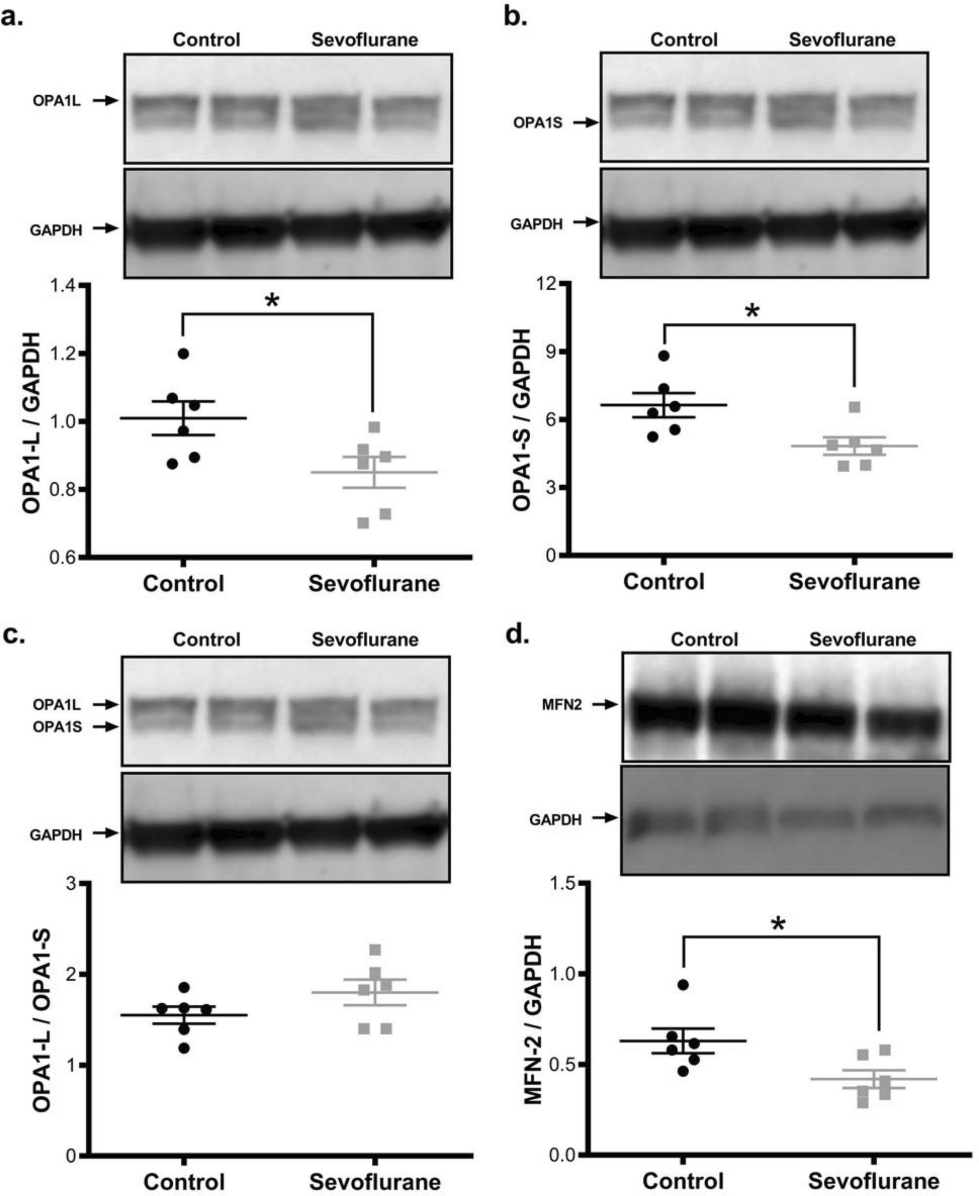
Protein mediators of mitochondrial fusion are reduced following P7 sevoflurane exposure. Pro-fusion proteins essential for inner- (OPA1 (**a**–**c**)), and outer- (MFN-2 (**d**)) mitochondrial membrane association are reduced in sevoflurane exposed tissue. While both the long and short forms of OPA1 were reduced, the ratio of long:short was increased, indicating an alteration to fusion activation. Values represent means ± SEM, with 6 animals in each group. Statistical significance between groups indicated with (*) for *p*-value < 0.05. All samples derived from same experiment and gels/blots processed in parallel.

To elucidate a mechanism by which sevoflurane exposure could alter mitochondrial morphology, we examined the level of proteins involved in mitochondrial fusion and fission. The primary driver of mitochondrial fission is the GTPase Dynamin-related Protein-1 (DRP1). We found that total DRP1 levels were reduced in sevoflurane treated brains, but that the ratio of activated (phospho-Ser616) to total DRP1 was profoundly increased (77% increase, *p* = 0.006), indicating a higher level of fission enzyme activity (Fig. 2a,b). DRP1 does not make physical contact with the mitochondrial membrane, rather it relies on adapter proteins to facilitate membrane cleavage. We found that inhalational anesthesia exposure significantly elevated the level of Mitochondrial Fission Factor (MFF) (60% increase, *p* = 0.04), the DRP1 adapter primarily responsible for membrane cleavage under cell stress (Fig. 2c). Since mitochondrial morphology is a balance between fusion and fission, we also examined proteins involved in the fusion process. We found that the two main fusion proteins, OPA1 (inner membrane) and MFN2 (outer membrane), have reduced levels after sevoflurane exposure (with a reduction in both the short (OPA-S) and long (OPA-L) forms of OPA-1) (Fig. 3). The reduction in OPA1-L is consistent with the observation of impaired cristae structure, as this protein is vital in maintenance of cristae folding and architecture^25^. These changes collectively represent a long-term shift in the mitochondrial morphological regulatory network, towards a fragmented mitochondrial phenotype in the sevoflurane exposed animals.

### Early sevoflurane exposure adversely affects adult mitochondrial protein homeostasis, organelle biogenesis, and oxidative phosphorylation

**Figure 4:**
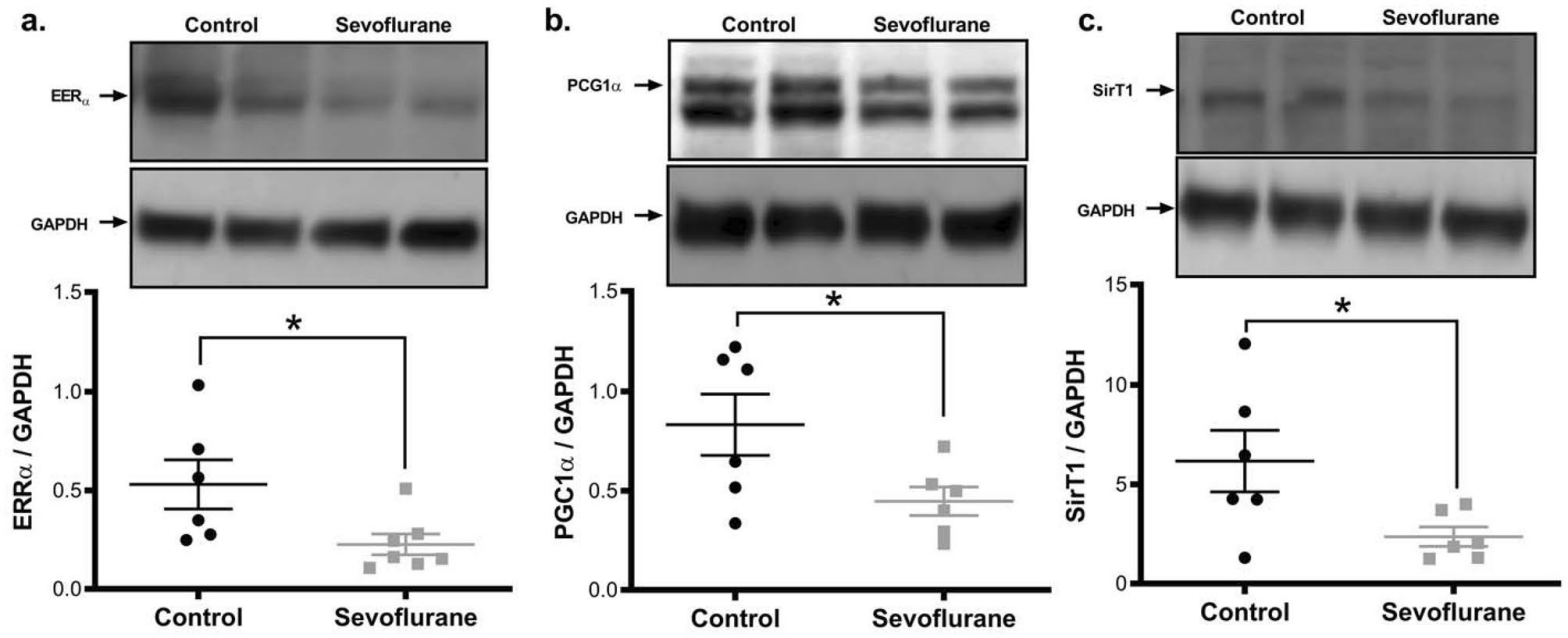
Adult cortical tissue demonstrates a lower level of mitochondrial biogenic regulators following infant sevoflurane exposure. Western blot analysis indicates sevoflurane exposed samples had a decrease in transcription factors responsible for mitogenic signaling/mitochondrial biogenesis (ERRα (**a**), PGC1-α (**b**)) and transcriptional regulation (SirT1 (**c**)), consistent with the reduction of cellular mitochondrial content and quality control. Values represent means ± SEM, with 6 animals in each group. Statistical significance between groups indicated with (*) for *p*-value < 0.05. All samples derived from same experiment and gels/blots processed in parallel.

To determine if the changes we observed in organelle morphology were accompanied by alterations to mitochondrial activity, we interrogated metrics commonly associated with the chronic mitochondrial dysfunction as typically observed in chronic neurological diseases and aging^26^. We found that mitochondria from animals exposed to inhalational anesthesia in infancy had a higher proportion of mitochondrial protein components encoded by nuclear genes (i.e. succinate dehydrogenase subunit A, SDHA) compared to those encoded by the mitochondrial genome (i.e. mitochondrially encoded cytochrome C oxidase subunit 1, mtCO1) (Fig. 2d), a sign of altered mitochondrial proteostasis and synthetic function^26,27^. This finding was supported by a reduction of mitochondrial genome component *ND1* relative to nuclear *GAPDH* (a higher ΔCt value, indicating a lower mitochondrial DNA content) (Fig. 1d) and a reduction in mitogenic transcriptional regulators (SirT1, ERRα and PGC1-α) in the sevoflurane exposed group (Fig. 4). As obligate aerobes, neurons are particularly dependent on mitochondrial derived ATP as they are unable to supplement their energetic demand with glycolysis^28^. In the sevoflurane exposed rats, we found an elevation of the ADP/ATP ratio, indicating a reduction in the available cellular energy (Fig. 1e, Supplemental Figure 1).

**Figure 5:**
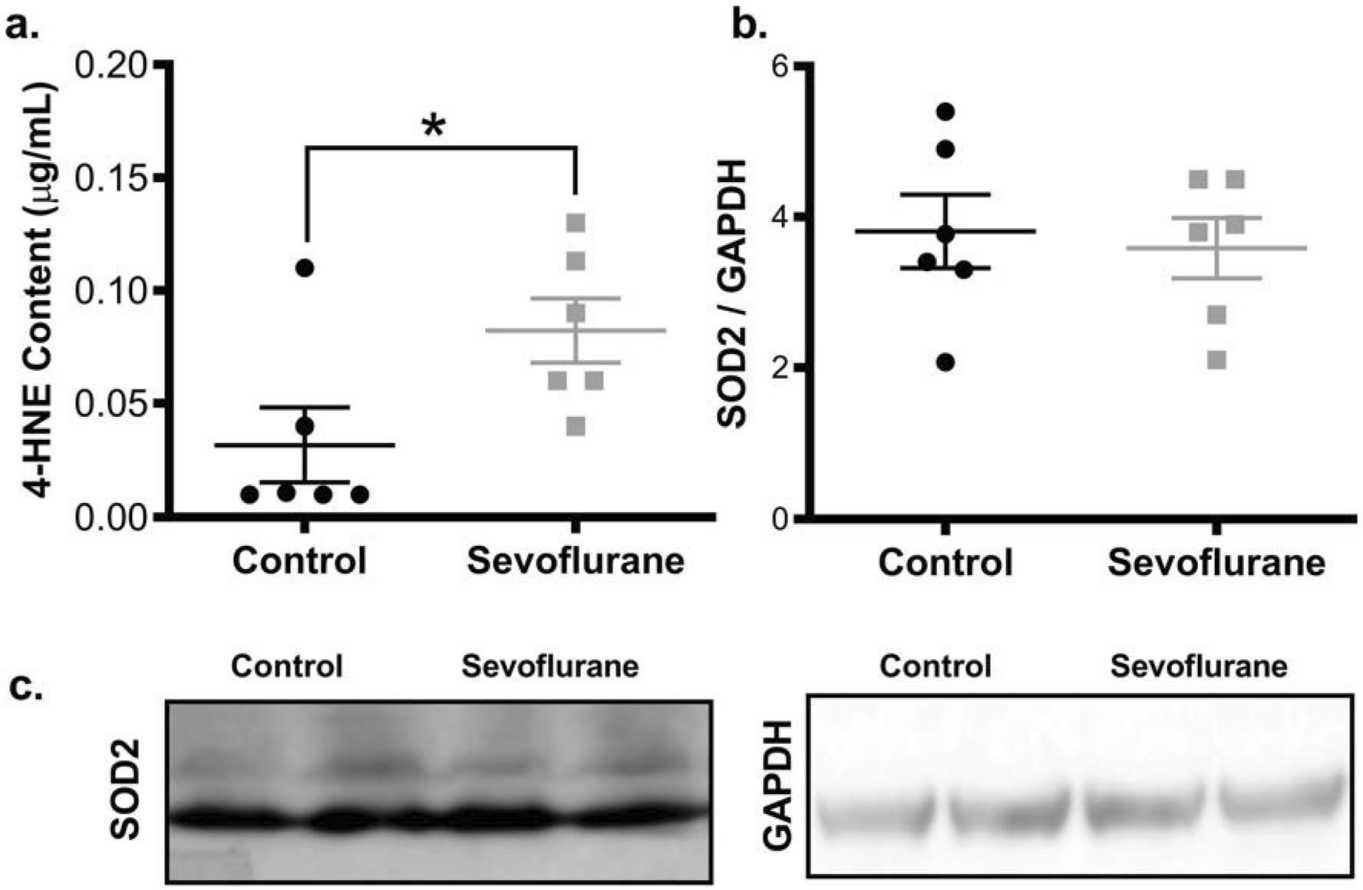
Adult rat cortex demonstrates long-term oxidative damage following P7 sevoflurane exposure. Lipid peroxidation adducts (4-HNE) were increased in sevoflurane exposed rat brains (**a**), indicating persistent oxidative stress. However, SOD2 has not increased, indicating the lack of a compensatory mitochondrial anti-oxidant response (**b**,**c**). Values represent means ± SEM, with 6 animals in each group. Statistical significance between groups indicated with (*) for *p*-value < 0.05. All samples derived from same experiment and gels/blots processed in parallel.

**Figure 6:**
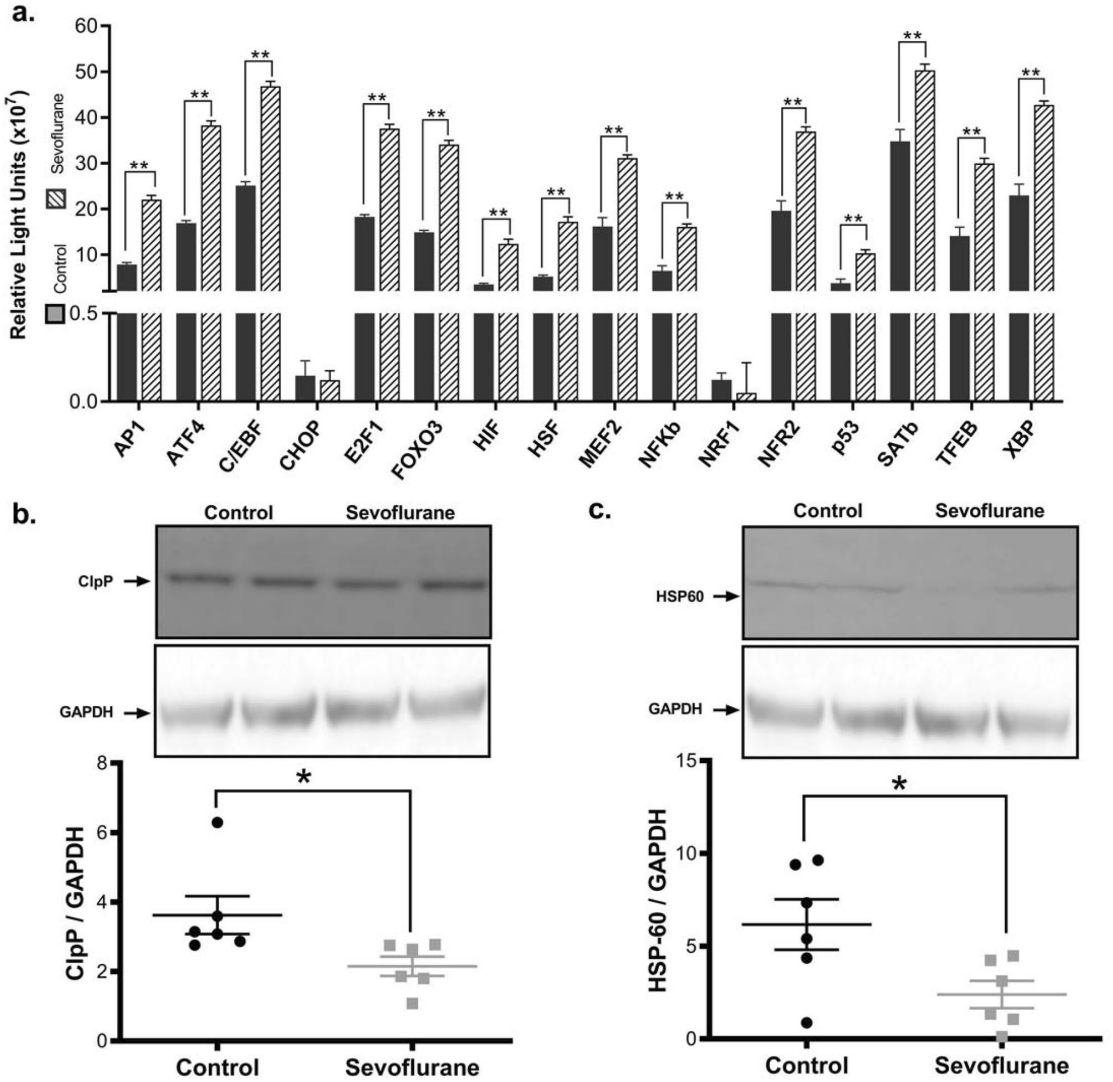
Sevoflurane exposure induces persistent alterations to the mitochondrial unfolded protein response (mtUPR). Quantification of nuclear-localized transcription factors reveals an increase in those responsible for mitochondrial stress response and mtUPR pathways after infant sevoflurane exposure (dashed bars), relative to non-exposed animals (black bars) (**a**). The observed changes in transcription factors were reflected in the levels of enzymes involved in the mitochondrial protein quality control (ClpP) (**b**) and folding (HSP-60) (**c**). Values represent means ± SEM, with 6 animals in each group. Statistical significance between groups indicated with (*) for *p*-value < 0.05, and (**) for *p-*value < 0.01.

Mitochondria are both a primary generator of ROS, and a main site of ROS-induced damage. In cortical tissue from animals with early sevoflurane exposure, we found a higher level of 4-HNE lipid adducts (1.9-fold increase, *p* = 0.04), a sign of chronic ROS damage (Fig. 5a), similar to those previously noted with acute anesthesia exposure^10,29^. As a corollary, we found elevated nuclear localization of transcription factors known to combat oxidative stress, including HIF, NFR2 and FOXO3 (Fig. 6a). Interestingly, these changes were not accompanied by an increase in the total level of SOD2, a mitochondrial anti-oxidant protein (Fig. 5b,c). Overall, our findings are consistent with disease states that induce chronic neuronal dysfunction, namely adverse changes to mitochondrial protein synthesis, energy production, mitochondrial biogenesis, and an elevation in long-term ROS generation and oxidative damage.

### Exposure to sevoflurane impairs the mitochondrial unfolded protein response (mtUPR)

The maintenance of proteostasis and the handling of misfolded proteins through the mitochondrial unfolded protein response (mtUPR) is an important determinant of mitochondrial health and sustained neuronal function. In sevoflurane-exposed animals, we found that 14 of 16 tested mtUPR-associated transcription factors had an enriched nuclear concentration, compared to unexposed animals (Fig. 6a). The increase in mtUPR- and mitochondria stress-associated transcription factors was accompanied by a decrease in measured end-effectors of the mtUPR, including HSP60 (61% reduction, *p* = 0.03) and ClpP (40% reduction, *p* = 0.04) (Fig. 6b,c). Interestingly, the decrease in HSP60 was in the context of a 3.2-fold increase in the nuclear concentration of the transcription factor responsible for its expression (HSF), illustrating significant problems with the governance of this quality control and stress response pathway. Dysregulation of the mtUPR is consistent with our observed mitonuclear protein imbalance described above^26^.

### Sevoflurane exposure increases neuroinflammatory mediators

**Figure 7:**
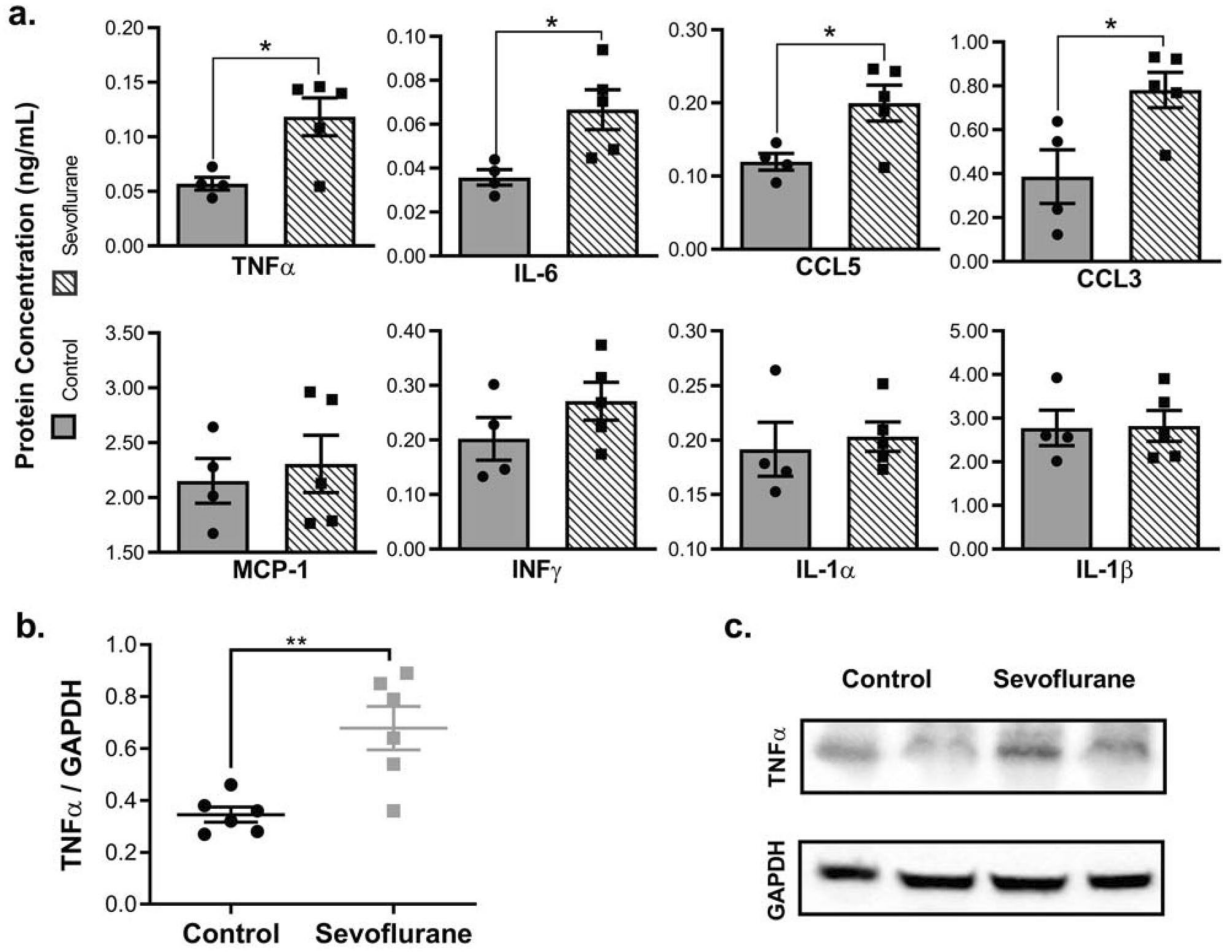
Early developmental exposure to sevoflurane increases the abundance of inflammatory cytokines in cortical rat tissue. Sevoflurane exposure increased the level of neuroinflammatory markers by ELISA (**a**) and Western blot (**b**,**c**), compared to controls. Values represent means ± SEM, with 6 animals in each group. Statistical significance between groups indicated with (*) for *p*-value < 0.05, and (**) for *p-*value < 0.01.

Neuroinflammation is a feature common in many disease states with chronic neuronal dysfunction^30^. To determine the potential role of neuronal inflammation in our model, we quantified the amount of inflammatory cytokines in sevoflurane exposed and control cortical tissue using an ELISA. We observed significant increases in four pro-inflammatory cytokines: Interleukin 6 (IL-6), Tumor Necrosis Factor alpha (TNFα), CCL5, and Macrophage Inflammatory Protein (MIP/CCL3) in the sevoflurane-exposed rats (Fig. 7a). As validation for the ELISA, we also performed Western blot analysis with TNFα, providing confirmatory results (Fig. 7b,c).

### Sevoflurane exposure reduces autophagosomal clearance and apoptotic signalling

Previous reports investigating acute or subacute exposures to sevoflurane have found significant and widespread neuronal apoptosis^7^. We investigated whether apoptotic cell death, and associated organelle quality control pathways (autophagy), were altered when the point of analysis was distal to the anesthesia exposure. Contrary to previous findings, we did not observe significant differences in cleaved (activated) caspase-3 between control and exposed brains (Supplemental Figure 2B). We did find a significant decrease in active LC3 in sevoflurane-exposed animals (17% decrease, *p* = 0.04) (Supplemental Figure 2A), indicating a reduction in autophagic activation and flux. In line with these findings, we observed an increase in the secreted neurotrophic factor cleaved (mature) brain-derived neurotropic factor (mBDNF), with no overall change in the uncleaved protein (proBDNF), and no observable difference in the levels of MEF2-A or MEF2-C, as transcriptional regulators of neurodevelopment and neuronal survival (Supplemental Figure 3). These results indicate that, despite the observed adverse changes in mitochondrial and inflammatory markers with anesthetic exposure, there is no evidence of continued cell death and an actual decrease in the turnover of damaged organelles. These findings do not rule out an increase in apoptosis at the exact time of drug exposure.

## Discussion

**Figure 8:**
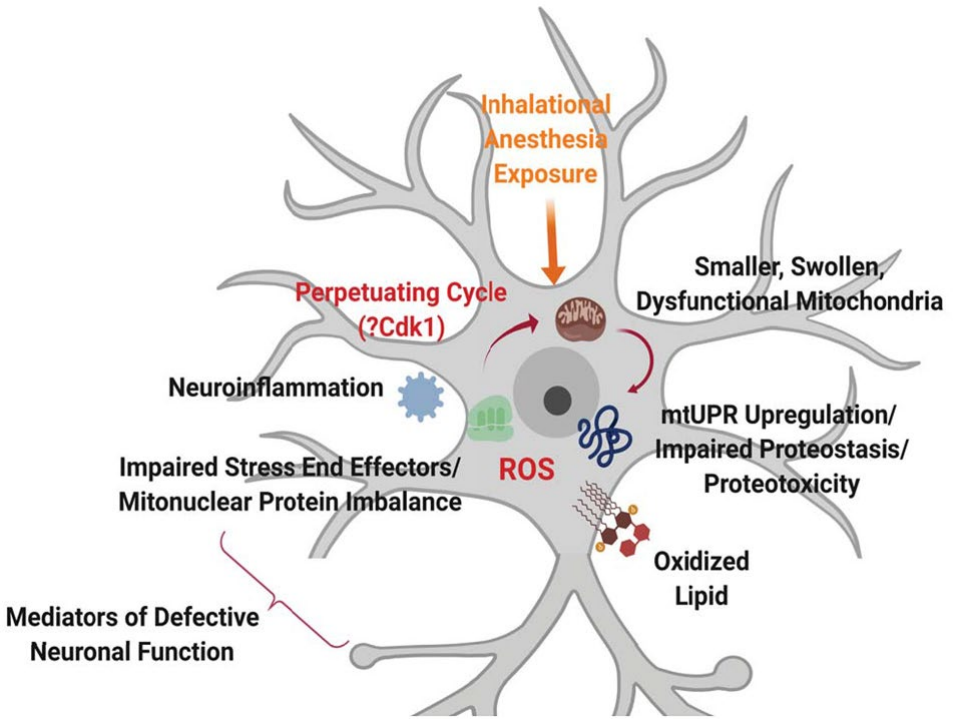
Early-life sevoflurane exposure can induce perpetuating neuronal dysfunction in the adult brain. Our findings indicate that anesthesia exposure in the developing brain adversely alters cellular energetic function through changes to mitochondrial morphology and function, resulting in a lasting adverse shift in mitochondrial quality control and regeneration/biogenesis pathways. This mechanism allows poorly functional organelles to persist, inducing proteotoxicity, oxidative stress, and neuroinflammation, creating a cell non-autonomous propagation of the phenotype, potentially through Cdk1.Image created using BioRender.com.

General anesthetics are widely administered to facilitate the safe completion of surgeries in the pediatric population, but concerns about their effects on the developing brain have led to a recent FDA warning. How anesthetic drugs may affect neuronal activity at a time distant to the drug exposure is not known. To address this question, we have utilized our rodent model of neurodevelopment and illustrated that sevoflurane exposure in infancy leads to adverse neurophysiological changes in the adult animal, including persistent changes to mitochondrial structure and function, protein quality control, increased oxidative stress, and neuroinflammation (Fig. 8). The observed alterations to cellular physiology reveal notable similarities with chronic neurodegenerative diseases, including a blunting of compensatory stress pathways, impairment of oxidative metabolism, impairments to mitochondrial biogenesis, and increased proteostasis/proteotoxicity.

Gas anesthetics are known to have an acute adverse effect on mitochondrial function, through multiple mechanisms, including the direct inhibition of mitochondrial respiratory chain activity^31^. We have described an additional chronic toxicity after an acute exposure, leading to a persistent stress state. Despite sevoflurane resulting in a lower cellular mitochondrial content, we found impaired mitogenic signalling with reductions in PGC1α, SirT1 and ERRα levels, considered essential compensatory mechanisms to rectify reduced mitochondrial productivity and to promote the genesis of new, healthy mitochondria^32^. At the protein level, we saw a shift in the mitochondrial fusion/fission balance, favoring smaller mitochondria, which are known to be less energetically efficient, as demonstrated by the increase in ADP/ATP ratio^33^. These smaller mitochondria would normally be cleared or repaired, but the observed decreased rate of autophagy would allow dysfunctional mitochondria to persist^34^. Mitochondrial architecture is intimately related to function, and our structural aberrations are logically consistent with the functional impairments described here and elsewhere^35^.

Despite significant increases in the transcription factors that regulate oxidative stress responses, protein misfolding pathways, and mitochondrial stress responses, we observed a lack of functional (proteomic) compensation in these areas. A lack of anti-oxidant response proteins in the face of oxidative stress has previously been observed following acute GA exposure, which led to the suggestion that SOD2 may represent a specific anesthesia target^10,29^. Alteration of the mtUPR is consistent with our previously published findings that illustrated how gas anesthetics alter protein folding and induce endoplasmic reticulum stress^36^. Many neurological disorders exhibit intracellular depositions of misfolded protein aggregates, and increases in mtUPR transcriptional regulators is one of the earliest phenotypic changes observed in chronic neurodegenerative diseases^37^. In Parkinson’s disease models, chronic activation of the mtUPR results in a time-dependent increase in mitochondrial dysfunction^38^. Similarly, we find that the nuclear transcriptional response is congruent with a stressed environment (retrograde response), but the resultant mitochondrial proteome (anterograde response) is inadequately protective^39^.

We did not observe any significant differences in cortical neuronal density or active apoptosis between control and sevoflurane-exposed animals. Our findings are in contrast to the widespread and cell type non-specific apoptosis observed in in vitro and in vivo models of (sub)acute anesthesia exposure (reviewed in^31^). It may be that apoptosis occurred in our model immediately after drug exposure, but without persistence into the adult brain where we still observe changes to cellular function, reducing the likelihood that apoptosis contributes significantly to the adult phenotype. It is possible that cell death during acute exposure could reduce brain development and neuronal connectivity, although organ plasticity and repair could compensate for such effects. In line with this idea, previous reports have indicated that when cognitive phenotypes are directly correlated to measured neuronal apoptosis, the phenotype can recover within days^40^. In isolated mouse neurons exposed to isoflurane, apoptosis was induced through an increase in proBDNF (p75^NTR^ activation), at the expense of neurotrophic mature BDNF levels (TrkB activation)^41^. Conversely, we found an increase in mature BDNF with sevoflurane exposure, possibly explaining the lack of apoptosis. While a pro-survival environment might be adaptive, perpetuating the existence of dysfunctional cells could be disadvantageous for developing synapse architecture.

Our observed molecular alterations parallel those found in common neurodegenerative disorders. This similarity is intriguing, as a potential relationship exists between general anesthetic exposure and the age of onset of neurodegenerative disease, though this association remains an area of active investigation^42,43^. Neuroinflammation is a hallmark of chronic neurodegenerative disease, with common elevations of IL-6 and TNFα, two cytokines increased in our samples. We observed significant elevation of the chemokine CCL5, previously illustrated to be elevated in Alzheimer’s and Parkinson’s, with a linear correlation to clinical severity in the latter disease^44^. The cytokine elevations observed in sevoflurane exposed animals are consistent with the increase in the nuclear localization of the NFR2 and XBP transcription factors (Fig. 6a), involved in inflammation-mediated stress mitigation responses^45^. The mitochondrial fragmentation exhibited in neurodegenerative disorders is usually associated with an increase in DRP1 activity, suggesting a common pathway related to mitochondrial morphology. The activating phosphorylation on DRP1 that we detected, pSer616, is a Cdk1-dependent site, a kinase known to be involved in the onset and evolution of neurodegenerative diseases^28^. Inflammatory cytokines can activate Cdk1, but their production is also regulated by the kinase, creating a cell non-autonomous method to expand dysfunctional signalling^46^. We postulate that anesthesia-induced metabolic and mitochondrial dysfunction could result in persistent upregulation of stress pathways, producing a vicious cycle of self-propagating neuroinflammation and further metabolic deficiency (Fig. 8). This includes persistent upregulation of DRP1 phosphorylation, production of inflammatory cytokines, and a deficiency in oxidative phosphorylation with an increase in ROS. Using these pathways, an early exposure to a GA could promote continued neuronal dysfunction and result in a persistent cognitive phenotype, similar to neurodegenerative diseases where chronic levels of misfolded proteins result in energetic failure and cellular oxidative stress. Gas general anesthetics may induce a similar proteotoxic stress with a self-perpetuating cycle of damage, inflammation and neuronal dysfunction. Further work is needed to determine if this pathway is unique, or only more noticed, in the developing brain.

The obligate dependence of neurons on mitochondria to meet their high energy demand makes neuronal activity particularly suspectable to mitochondrial dysfunction. While the results presented here are discussed in this context, it is important to note that our methodology precludes the ability to attribute all findings specifically to effects on neurons. Adverse impact on other cell types, most notably glial cells which have a high abundance and functional role in the cortex, likely also contributes to the phenotype.

In conclusion, our results indicate that exposure to sevoflurane during a developmentally sensitive period results in persistent cortical impairment through the induction of molecular changes consistent with an energy deficient state, neuroinflammation and altered proteostasis, without proper compensatory responses. Our study supports the idea that therapeutic agents aimed to reduce energetic dysfunction at the time of surgery may represent viable options to mitigate developmental toxicity, and that markers of neuroinflammation may be useful to acutely determine anesthetic effects.

## Materials and methods

### Experimental animals

Experiments were performed using male Sprague Dawley rats (total N = 12), randomly assigned to experimental (sevoflurane exposed) and control groups. Experimental and control animals were equally co-populated and maintained in identical environments, including food and water, temperature and light/dark cycles, to the best of our ability. There was no animal mortality during the entire time of observation. All experiments were approved by the Institutional Animal Care and Use Committee of the University of California (San Francisco, California) and performed in accordance with national and institutional guidelines for animal care. All animal experiments were carried out according to the ARRIVE 2.0 guidelines, as described in the relevant section.

### Sevoflurane exposure

Anesthetic exposure was performed according to our previously described protocol^13^. Briefly, on postnatal day 7 (P7), rat pups were randomly assigned to either exposed to 1 minimum alveolar concentration of sevoflurane (corresponding to ~ 5% atm), or identical environment without anesthetic agent as sham controls (6 animals per group) for a total of 4 h (FiO_2_ = 0.5), as we performed previously^11,13,21,22,47^. P7 was used as the exposure time point as it represents the period of peak synaptogenesis and the most vulnerable period for GA mediated neurotoxicity in rodent models^48^.

### Animal euthanization and tissue collection

Ten months following anesthetic exposure (P300), all animals were euthanized following deep anesthetization with isoflurane (greater than 5% with loss of pedal pain reflex) by subsequent transcardial perfusions of 0.9% saline and 4% paraformaldehyde in 0.1 M phosphate buffered saline (pH 7.4). Cortical brain regions were immediately placed in preservation solution (0.21 M mannitol, 0.07 M sucrose and 20% DMSO) and flash frozen with ethanol and dry ice. Samples were stored at − 80 °C and shipped to the Hospital for Sick Children for downstream analysis. Following tissue collection, all experiments were performed blinded until grouping for analysis.

### Transmission electron microscopy (TEM)

All TEM for mitochondrial ultrastructural analysis was performed by the Pathology Lab facility at the Hospital for Sick Children, Toronto, Canada. Images were captured on a JEOL JEM1011 (JEOL, Inc., Peabody, MA) microscope. TEM images (20,000× magnification) were analyzed using unbiased automated object identification in ImageJ^49^. For mitochondrial size analysis, mitochondrial area was blindly quantified in a minimum of ten random microscope fields, per sample.

### Quantitative polymerase chain reaction (qPCR)

To analyze variations in mitochondrial content, we performed qPCR on isolated genomic DNA, as previously described^50^. Briefly, genomic DNA was isolated from 10 mg of cortical mouse tissue using Tissue Genomic DNA Mini Kit (Geneaid, New Taipei City, Taiwan), as per manufacturer’s instructions. Each 20 μL qPCR reaction contained 10 μL SsoFast EvaGreen PCR Supermix (Bio-Rad), 0.8 μL forward and reverse mitochondrial primers (10 μM) (amplifying the *ND1* region of the mitochondrial genome) (Supplemental Table 2), 3 μL genomic DNA (10 ng/μL), and sterile water to the final volume. Reactions were performed using a CFX96 Real Time PCR instrument (Bio-Rad), using CFX Manager Software to determine Cq values. Mitochondrial DNA content (*ND1*) was normalized to *GAPDH* using a ΔCq method, providing a relative cellular mitochondrial genome content^50^.

### ADP/ATP ratio assay

As an indicator of cellular energy status, ADP/ATP ratio was performed using the Abcam Bioluminescent ADP/ATP Ratio Assay Kit and performed according to manufacturer’s instructions (ab65313, Cambridge, MA, USA). In brief, 100 µL of ATP Reaction Mix (1× ATP Monitoring Enzyme in Nucleotide Releasing Buffer) was loaded on a 96-well plate, followed by a baseline plate reading. To measure ATP levels, 100 µg of cortical lysate was then added to each well, followed by 2 min incubation and plate read. Finally, to determine ADP levels, 100 µL ADP (1× ADP Converting Enzyme in Nucleotide Releasing Buffer) was then added to each well and incubated for 2 min, followed by a final plate reading. ADP and ATP content quantified using a standard curve. Bioluminescence measured on Varioskan LUX Plate Reader (Thermo Scientific, MA, USA).

### Western blots

All Western blots were performed on cortical lysate, which was prepared by placing 20 mg of tissue into a 1.5 mL microcentrifuge tube containing a 5 mm stainless steel bead (Qiagen), 300 μL cold 1× RIPA buffer and 1× cOmplete Protease Inhibitor Cocktail (Sigma-Aldrich). Samples were loaded into the TissueLyser II (Qiagen), and subjected to 2 cycles of agitation of 2 min at 20 Hz. Following homogenization, an additional 200 μL of RIPA was added, and samples were agitated for 2 h at 4 °C, followed by 20 min centrifugation at 15,000×*g*. Supernatants were collected, and protein content quantified using Pierce BCA Protein Assay Kit (Thermo Scientific). Cellular lysates were separated on a 4–12% (w/v) gradient SDS-PAGE gels (Genscript, Piscataway, NJ), before transfer to 0.2 μm PVDF membranes with a Trans-Blot Turbo Transfer System (0.8A and 25V) (Bio-Rad, Hercules, CA). Membranes were blocked (TBS-T + 5% skim milk) for 1 h, followed by overnight incubation with a primary antibody (TBS-T + 1% skim milk) at 4 °C (see Supplemental Table 1). Membranes were washed in TBS-T for 15 min prior to incubating in species specific horseradish peroxidase (HRP)-linked secondary antibody (TBS-T + 1% skim milk) for 1 h. Protein levels were determined by imaging on a Gel-Doc XRSystem (Bio-Rad) following detection with the Enhanced Chemiluminescence System (GE Life Sciences, Mississauga, Canada). The optical densities obtained were analysed with ImageJ, with GAPDH used as a loading control^49,51,52^. All samples derived from same experiment and gels/blots were processed in parallel.

### 4-Hydroxynonenal (HNE) adduct ELISA

To examine oxidative damage in the cortical tissue, we quantified the abundance of 4-HNE protein adducts using the OxiSelect HNE Adduct Competitive ELISA Kit, as per manufacturer’s specifications (Product STA-838-T, Cell BioLabs, San Diego, CA, USA). Briefly, ~ 500 μg of total protein from lysed cell extracts in RIPA buffer were plated on an HNE-conjugated ELISA plate, followed by the addition of an anti-HNE polyclonal antibody and HRP-conjugated secondary antibody. The HNE adduct content was quantified spectrophotometrically at 450 nm, with comparison to an HNE-BSA standard curve, and normalized to total protein loading.

### Mitochondrial unfolded protein response (mtUPR) profile

To quantify mtUPR activation, we determined the nuclear localization of mtUPR-related transcription factors (TF) using the Mitochondrial UPR TF Activation Profiling Plate, as per manufacturer’s instructions (including AP1, ATF4, C/EBP, CHOP, E2F1, FOXO3, HIF, HSF, MEF2, Nfκβ, NFR1, NFR2/ARE, p53, SATB, TFEB and XBP) (Product FA-1006, Signosis, Santa Clara, CA, USA). Briefly, nuclear extracts were isolated from 50 mg of brain tissue using the Nuclear Extraction Kit (Signosis). Following isolation, 15 μg of nuclear extracts were mixed with biotin-labeled DNA probes (containing TF consensus sequences) resulting in TF:DNA probe complexes. Following spin-column purification, DNA probes were removed, and the resulting TF extracts were added to a pre-coated plate with TF specific complementary sequences. TF content was detected with streptavidin-HRP conjugate.

### Inflammatory cytokine ELISA

Inflammatory cytokines were quantified using the Rat Inflammation ELISA Strip Assay, as per manufacturer’s instructions (Product EA-1201, Signosis, Santa Clara, CA). Briefly, cortical tissue was lysed in Lysis Buffer and 10 µg total protein loaded into wells coated with primary antibodies against inflammatory cytokines (TNFα, IL-6, IL-1α, IL-1β, IFNγ, MCP-1, CCL3 and CCL5). Following incubation with enzyme-linked antibodies, HRP substrate was added to produce a colorimetric response, which was quantified spectrophotometrically at 450 nm. Cytokine quantification was derived using a standard curve (Product EA-1202, Signosis).

### Statistical analysis

Unless otherwise noted, all results are presented as means ± SEM. Following a Kolmogorov–Smirnov test for normalcy, statistical significance was determined using either un-paired Student’s t-test with Welsh correction or un-paired Mann–Whitney U-test, as appropriate. Where more than one comparison was performed, significance was determined using an ANOVA, using the Dunnett method for multiple comparison correction. Statistical significance was calculated using GraphPad Prism 6.0 using a *p*-value of < 0.05 as a cut-off for significance.

## Supplementary Information

Supplementary Information.
