# Supplementary material for: Singular and short-term anesthesia exposure in the developing brain induces persistent neuronal changes consistent with chronic neurodegenerative disease
Source: Sci Rep. 2021 Mar 11;11:5673. doi: 10.1038/s41598-021-85125-5 (PMC7952562; doi:10.1038/s41598-021-85125-5)
Supplement: Supplementary file 1 — Supplementary Information. [file 41598_2021_85125_MOESM1_ESM.pdf]

SUPPLEMENTARY MATERIALS

SINGULAR AND SHORT-TERM ANESTHESIA EXPOSURE IN THE DEVELOPING BRAIN INDUCES PERSISTENT NEURONAL CHANGES CONSISTENT WITH CHRONIC NEURODEGENERATIVE DISEASE

Kaley Hogarth[1,2], Ramesh Babu Vanama, PhD[1,2], Greg Stratmann, MD, PhD[3], Jason T Maynes, PhD/MD[1,2,4,*]

[1]Division of Molecular Medicine, SickKids Research Institute, Toronto, Canada.

[2]Department of Anesthesia and Pain Medicine, Hospital for Sick Children, Toronto, Canada

[3]Department of Anesthesia and Perioperative Medicine, University of California, San Francisco, San Francisco, USA

[4]Department of Anesthesia, University of Toronto, Toronto, Canada

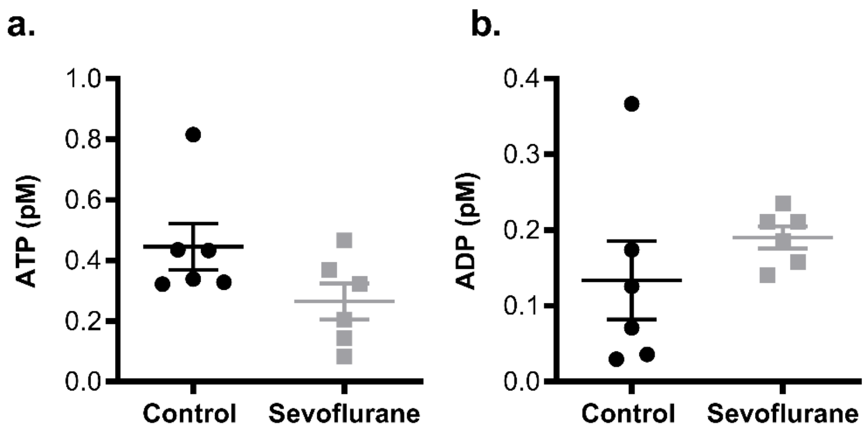

Supplemental Figure 1. Infant exposure to sevoflurane alters cellular energetic status. Sevoflurane exposed samples demonstrated trends in both a decrease in ATP (**a)** and increase in ADP (**b**). Values represent means ± SEM, with 6 animals in each group.

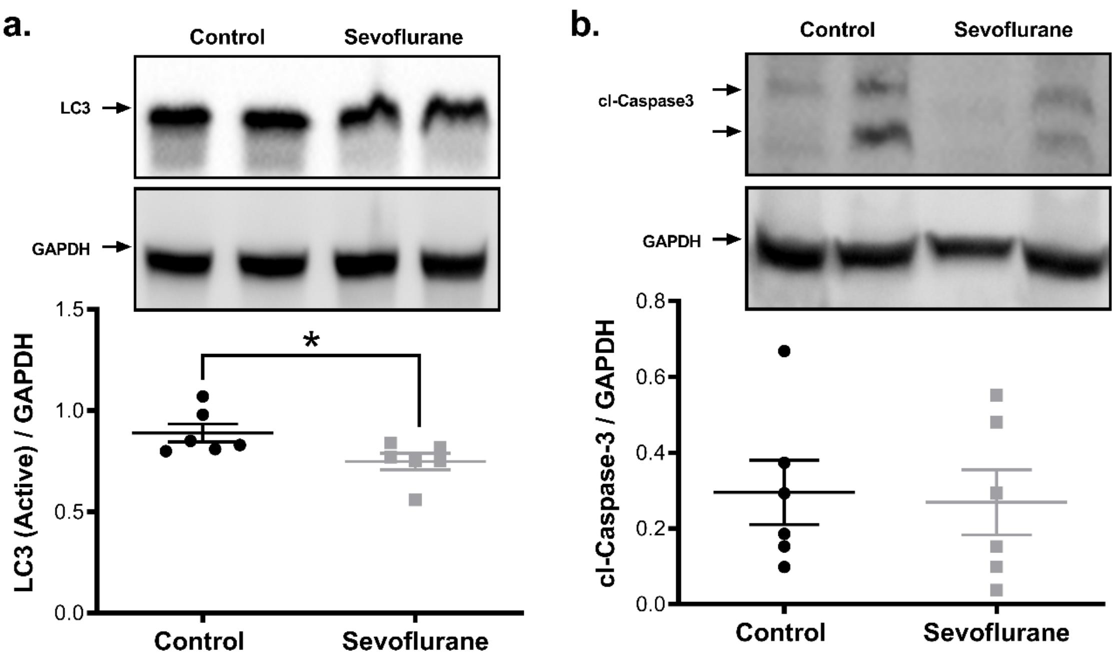

Supplemental Figure 2. Infant exposure to sevoflurane alters autophagosome activation but not apoptotic signaling in adult rat cortex. Sevoflurane exposed samples demonstrated a slight, but significant, increase in autophagosome formation (activated isoform of LC3)(**a**), but no significant activation of apoptosis, as measured by changes to the pro-apoptotic form of caspase-3 (cl-caspase-3) (**b**). Values represent means ± SEM, with 6 animals in each group. Statistical significance between groups indicated with (*) for *p*-value <0.05.

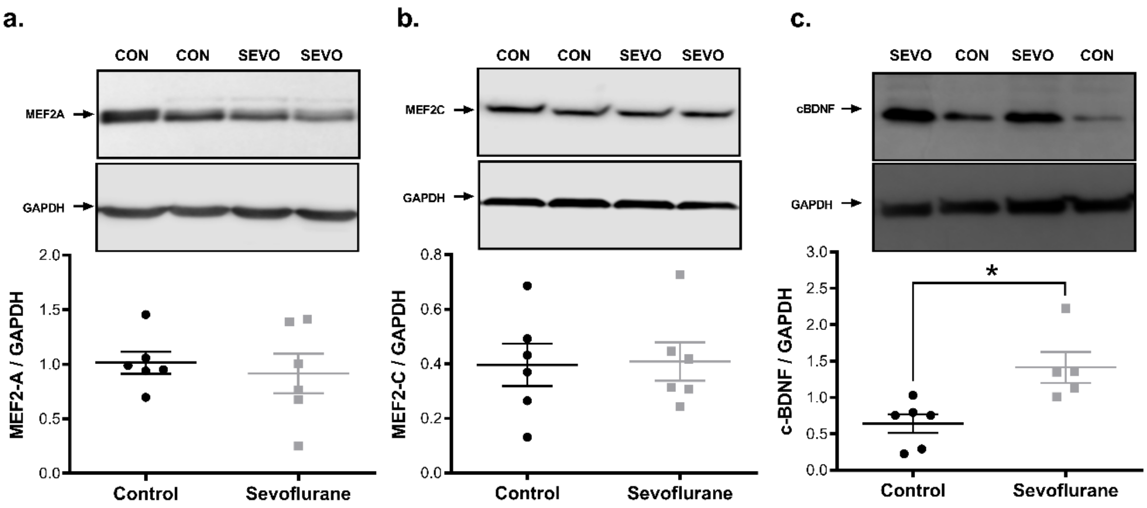

Supplemental Figure 3. Sevoflurane does not alter the total level of neurogenic transcription factors but does affect neurotropic signaling. While sevoflurane exposed rats (SEVO) demonstrated no significant changes to total expression levels of BDNF or MEF2 isoforms (**a, b**) compared to controls (CON), exposure did result in an increase in the cleaved BDNF (c-BDNF) (**c**) indicating a shift in the balance between functionally distinct isoforms. Values represent means ± SEM, with 6 animals in each group. Statistical significance between groups indicated with (*) for *p*-value <0.05.

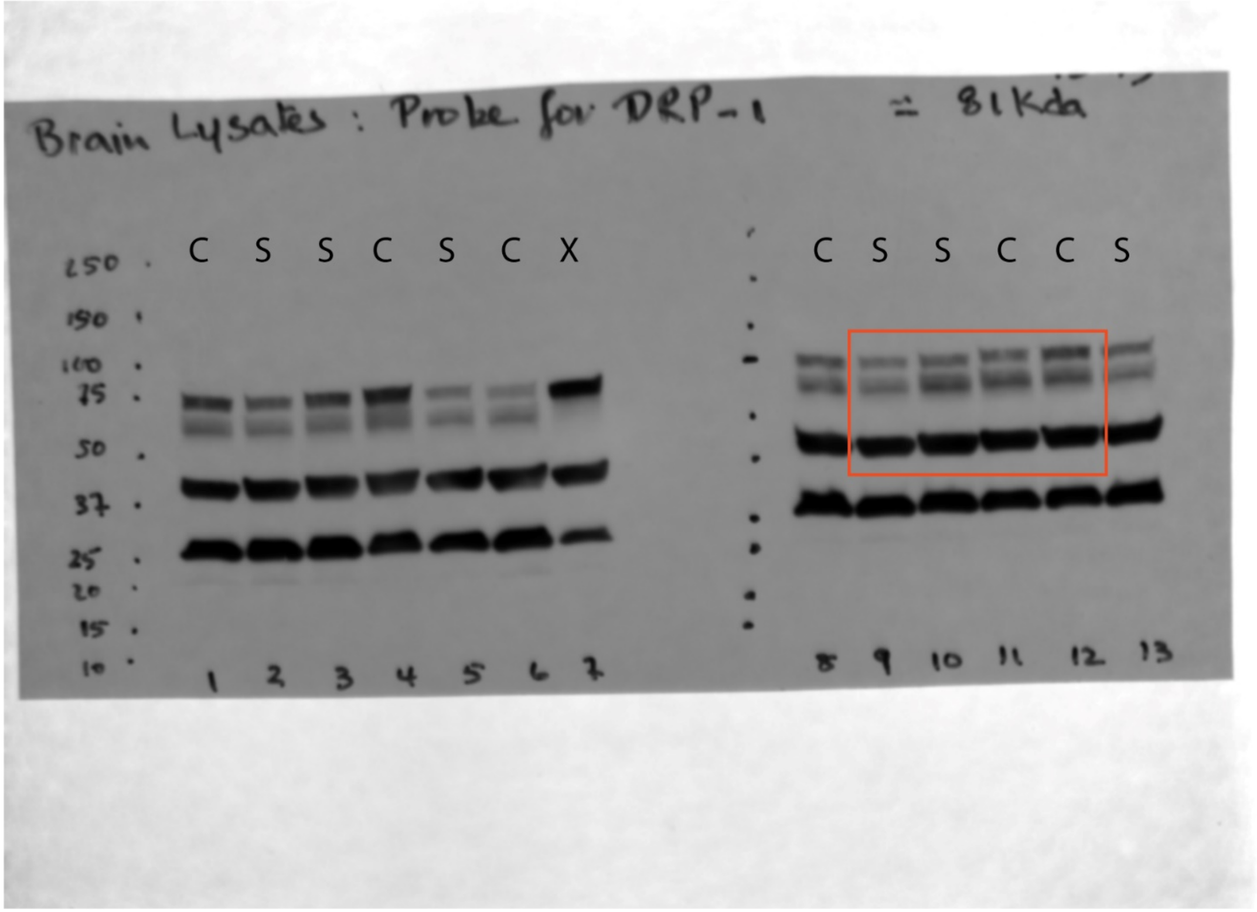

Supplemental Figure 4: Full length membranes used in Western blot analysis of DRP1. Membranes probed with DRP1, and GAPDH as a loading control. Red box denotes representative bands used in the main figure image. Labels on each lane indicate sevoflurane exposed (**S**) and control (**C**) samples. All samples were derived from same experiment, with gels/blots processed in parallel. Blinding during analysis is illustrated by the altered ordering of control/sevoflurane samples. An internal sample control is included (“X” marked).

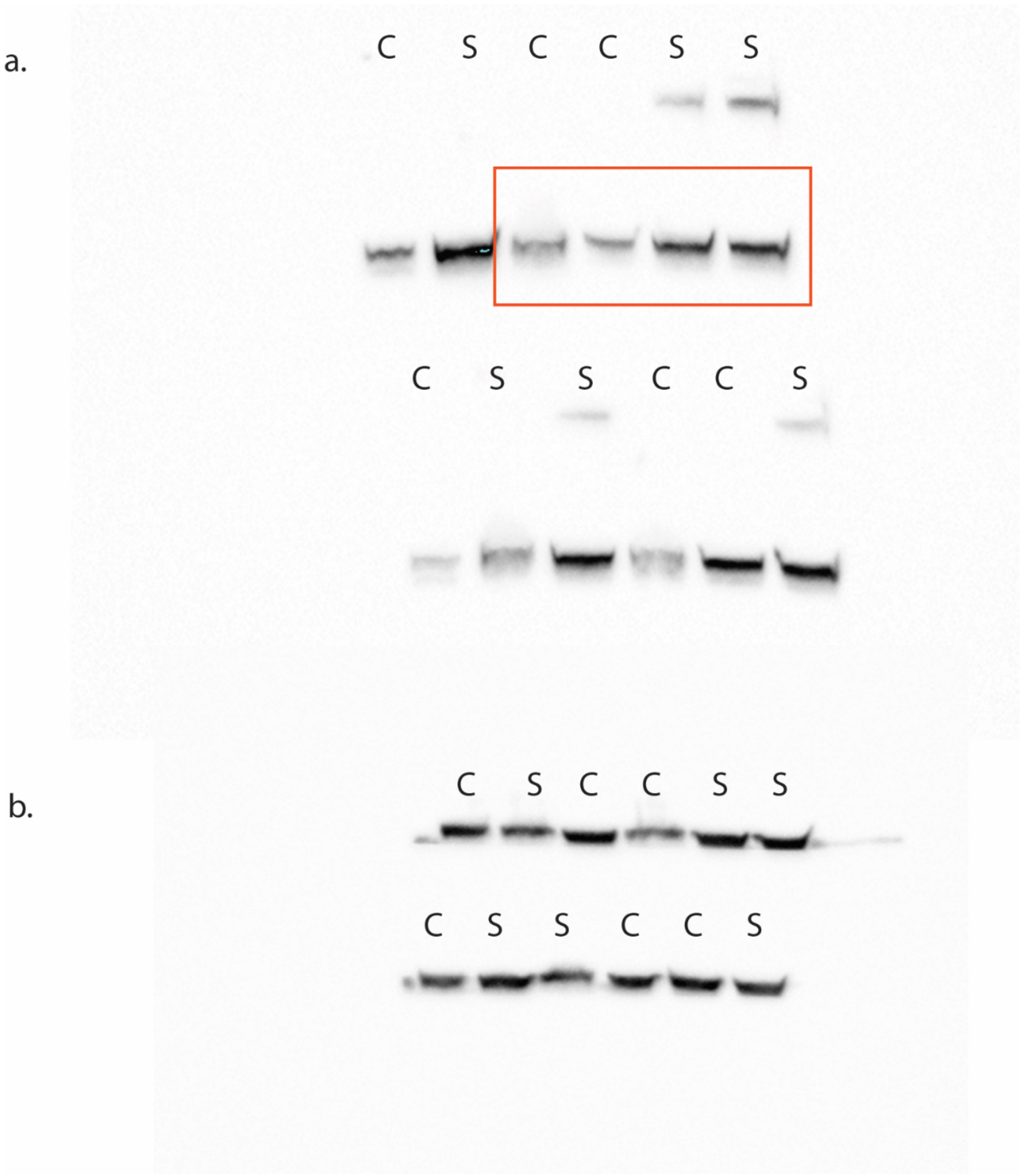

Supplemental Figure 5: Full length membranes used in Western blot analysis of p-DRP1. Membranes probed with (**a**) p-DRP1, and (**b**) GAPDH as a loading control. Red box denotes representative bands used in the main figure image. Labels on each lane indicate sevoflurane exposed (**S**) and control (**C**) samples. All samples were derived from same experiment, with gels/blots processed in parallel. Blinding during analysis is illustrated by the altered ordering of control/sevoflurane samples.

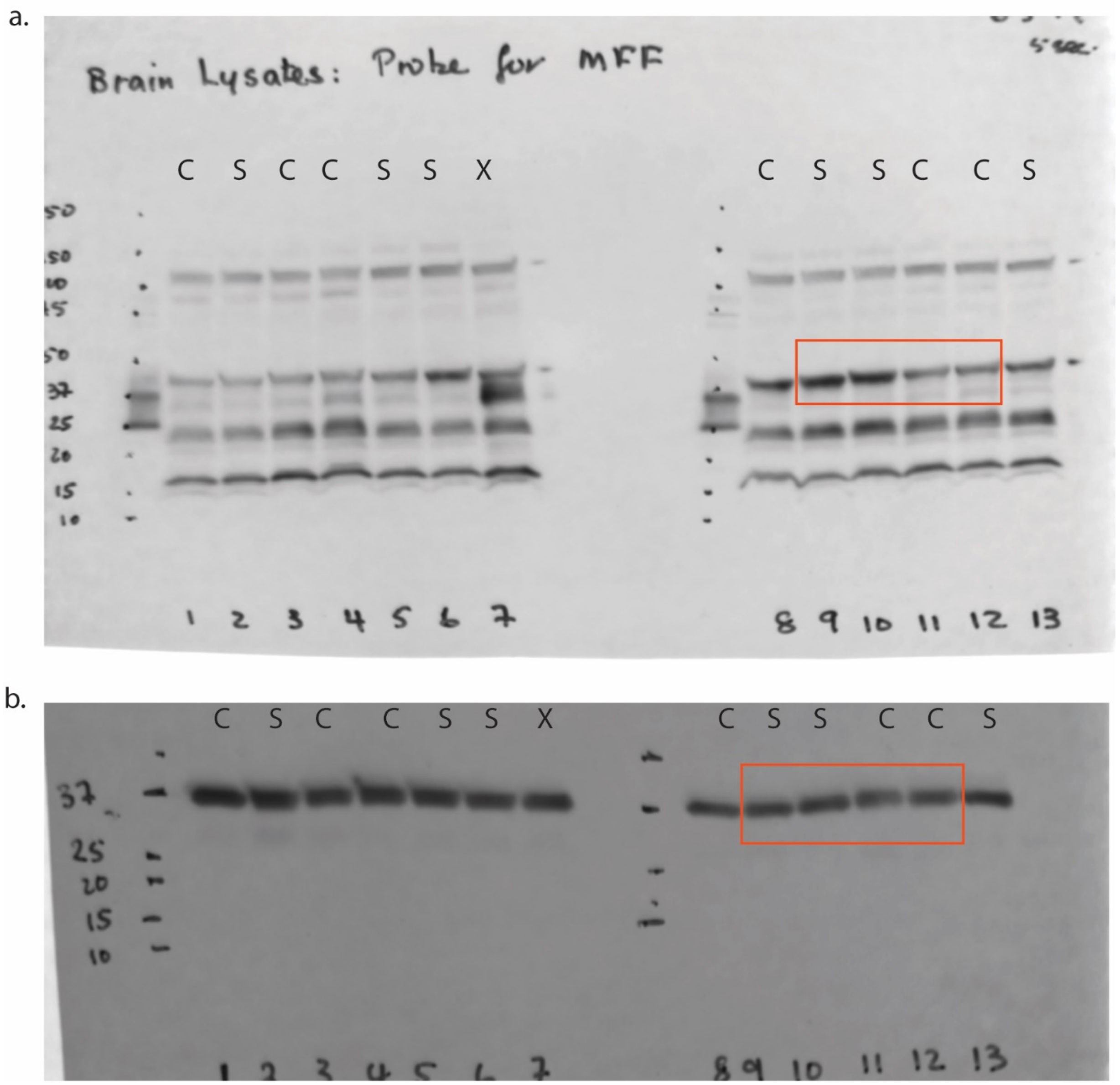

Supplemental Figure 6: Full length membranes used in Western blot analysis of MFF. Membranes probed with (**a**) MFF, and (**b**) GAPDH as a loading control. Red box denotes representative bands used in the main figure image. Labels on each lane indicate sevoflurane exposed (**S**) and control (**C**) samples. All samples were derived from same experiment, with gels/blots processed in parallel. Blinding during analysis is illustrated by the altered ordering of control/sevoflurane samples. An internal sample control is included (“X” marked).

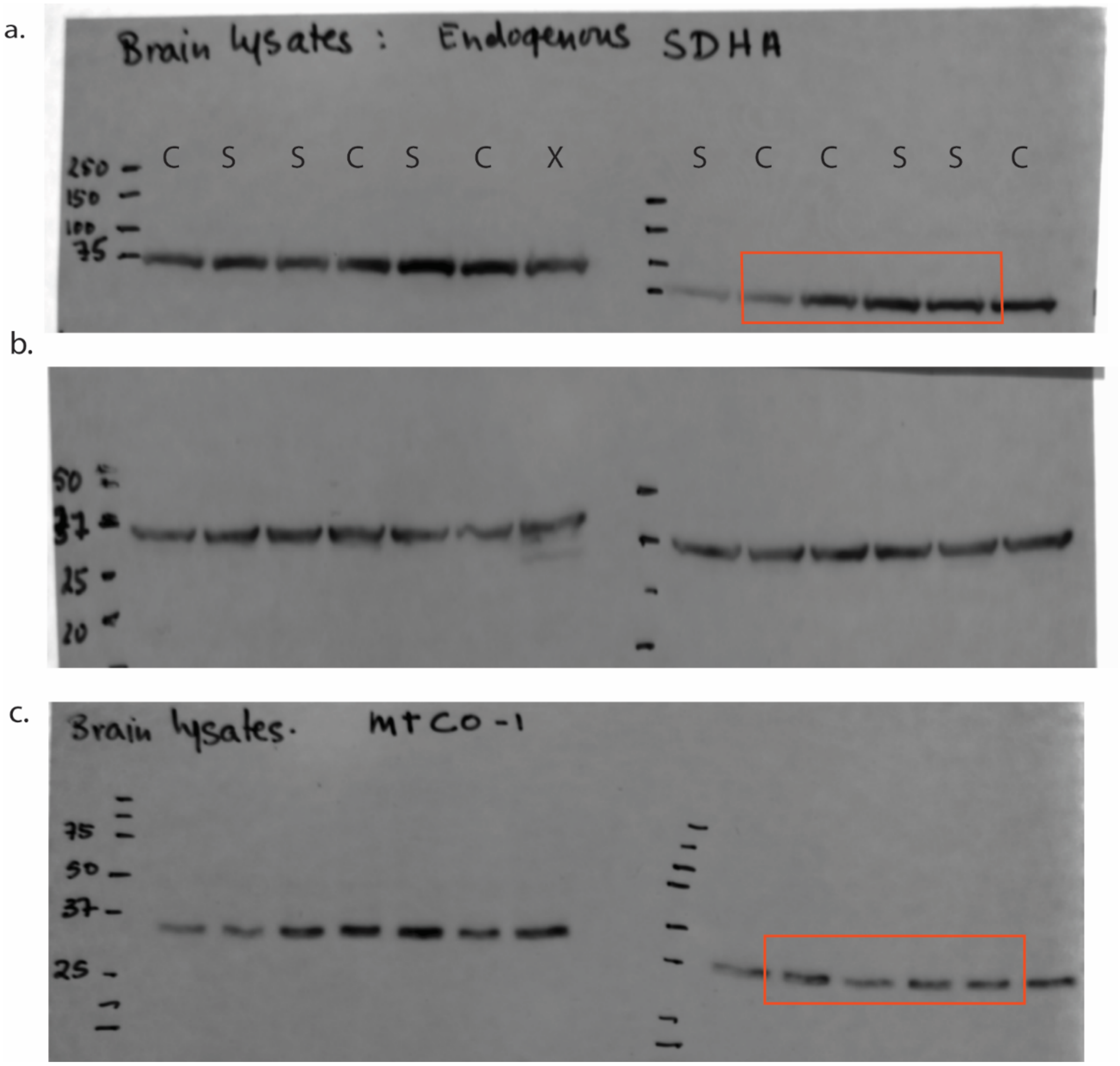

Supplemental Figure 7: Full length membranes used in Western blot analysis of SDHA and mtCO1. Membranes probed with (**a**) SDHA, (**c**) mtCO1, and (**b**) GAPDH as a loading control. Red box denotes representative bands used in the main figure image. Labels on each lane indicate sevoflurane exposed (**S**) and control (**C**) samples. All samples were derived from same experiment, with gels/blots processed in parallel. Blinding during analysis is illustrated by the altered ordering of control/sevoflurane samples. An internal sample control is included (“X” marked).

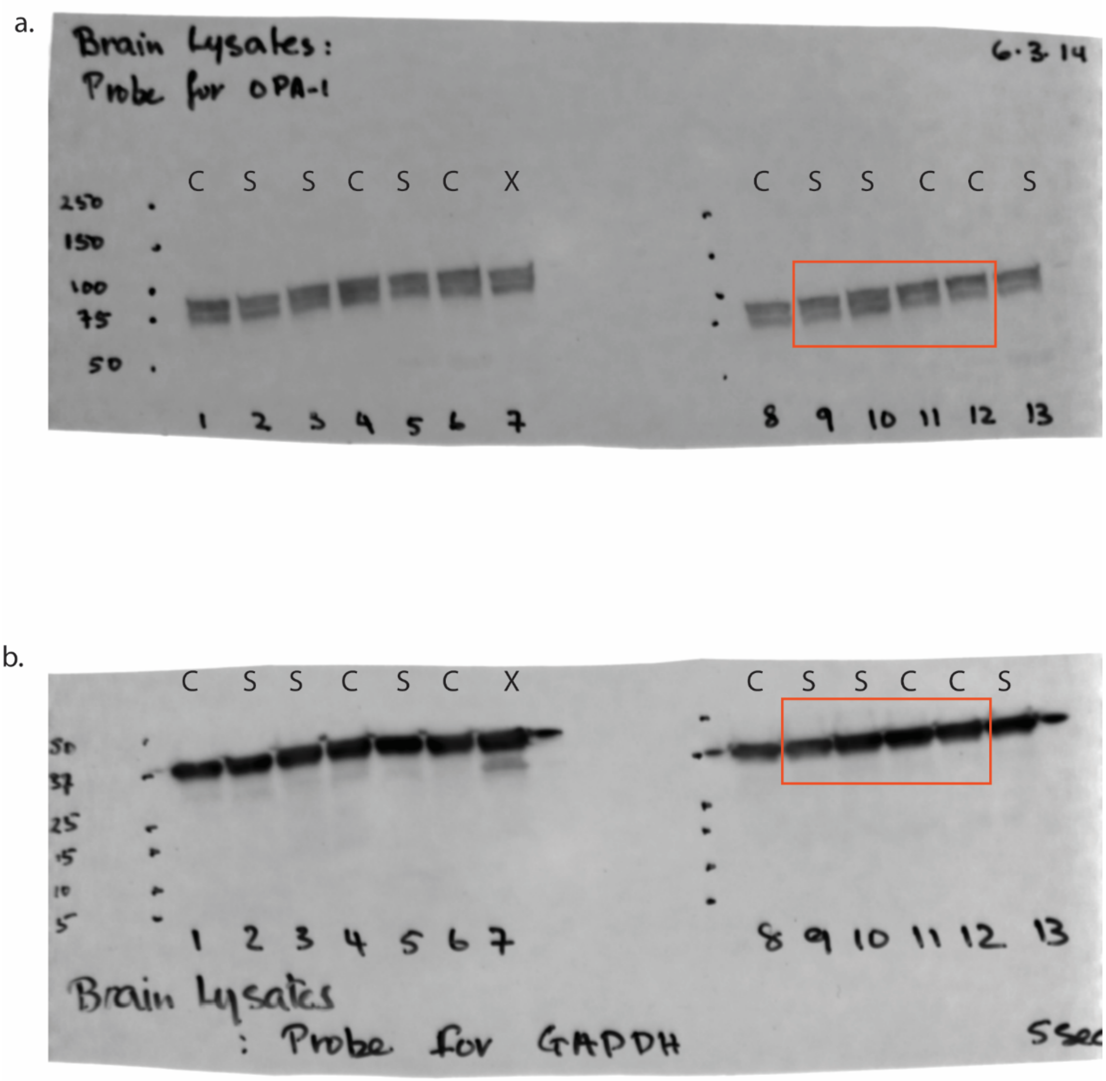

Supplemental Figure 8: Full length membranes used in Western blot analysis of OPA1. Membranes probed with (**a**) OPA1 (both, long (OPA1L) and short (OPA1S) isoforms) and (**b**) GAPDH as a loading control. Red box denotes representative bands used in the main figure image. Labels on each lane indicate sevoflurane exposed (**S**) and control (**C**) samples. All samples were derived from same experiment, with gels/blots processed in parallel. Blinding during analysis is illustrated by the altered ordering of control/sevoflurane samples. An internal sample control is included ("X" marked).

a.

b.

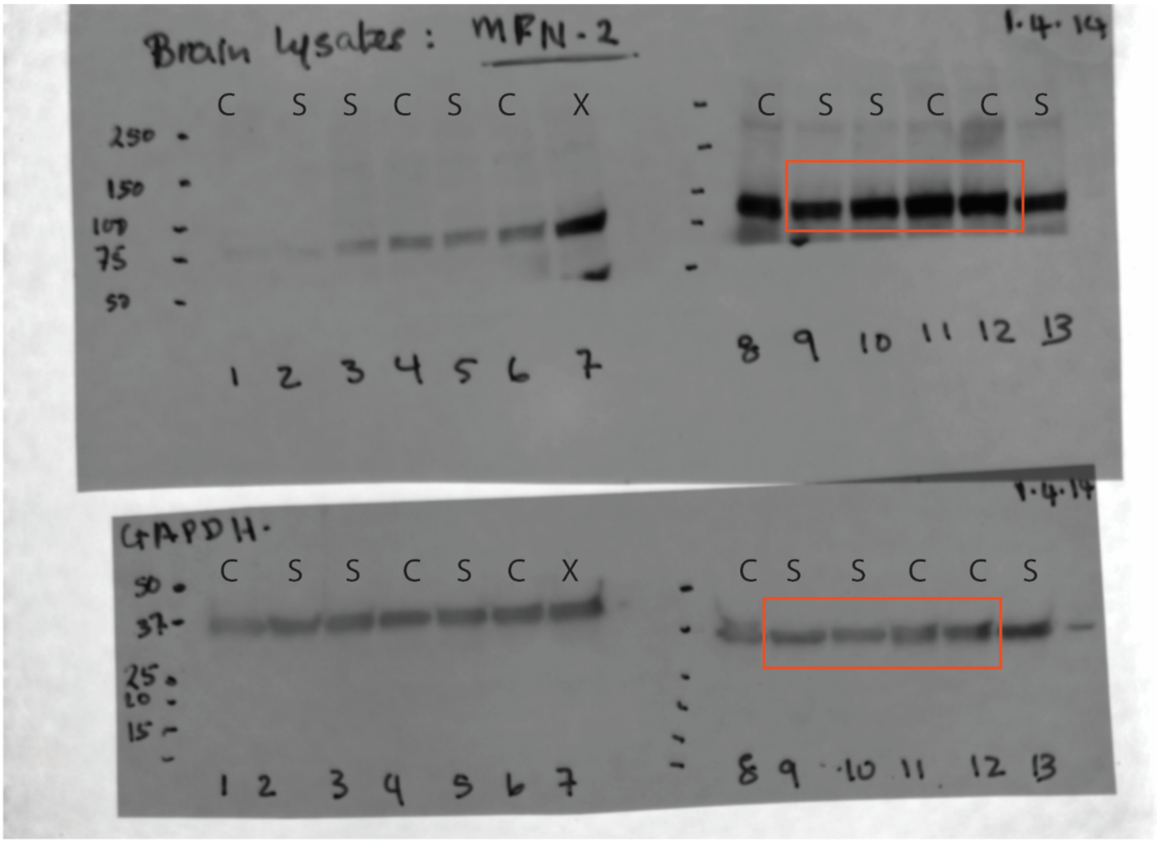

Supplemental Figure 9: Full length membranes used in Western blot analysis of MFN2. Membranes probed with (**a**) MFN2 and (**b**) GAPDH as a loading control. Red box denotes representative bands used in the main figure image. Labels on each lane indicate sevoflurane exposed (**S**) and control (**C**) samples. All samples were derived from same experiment, with gels/blots processed in parallel. Blinding during analysis is illustrated by the altered ordering of control/sevoflurane samples. An internal sample control is included ("X" marked).

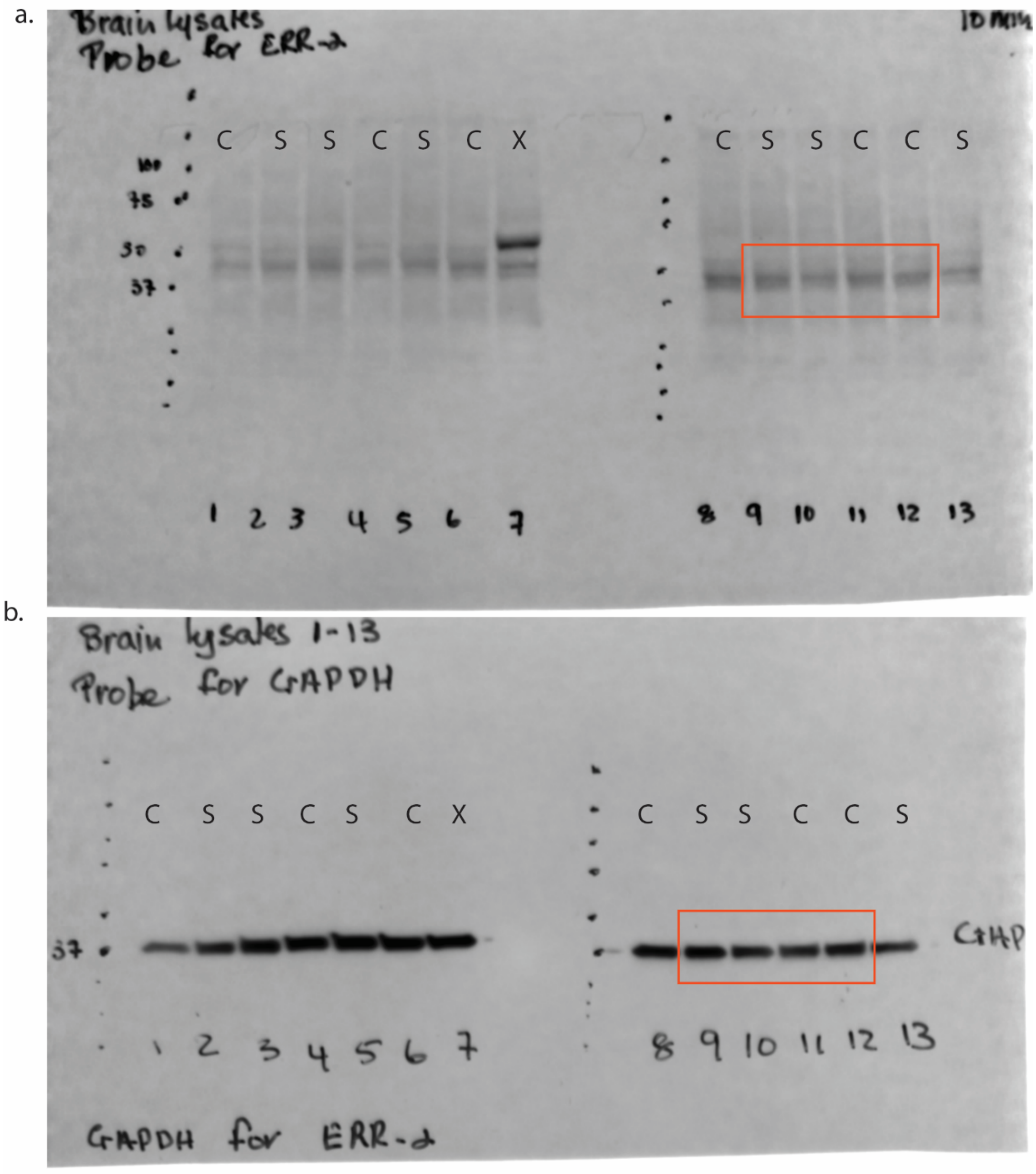

Supplemental Figure 10: Full length membranes used in Western blot analysis of ERRα. Membranes probed with (**a**) ERRα and (**b**) GAPDH as a loading control. Red box denotes representative bands used in the main figure image. Labels on each lane indicate sevoflurane exposed (**S**) and control (**C**) samples. All samples were derived from same experiment, with gels/blots processed in parallel. Blinding during analysis is illustrated by the altered ordering of control/sevoflurane samples. An internal sample control is included ("X" marked).

a.

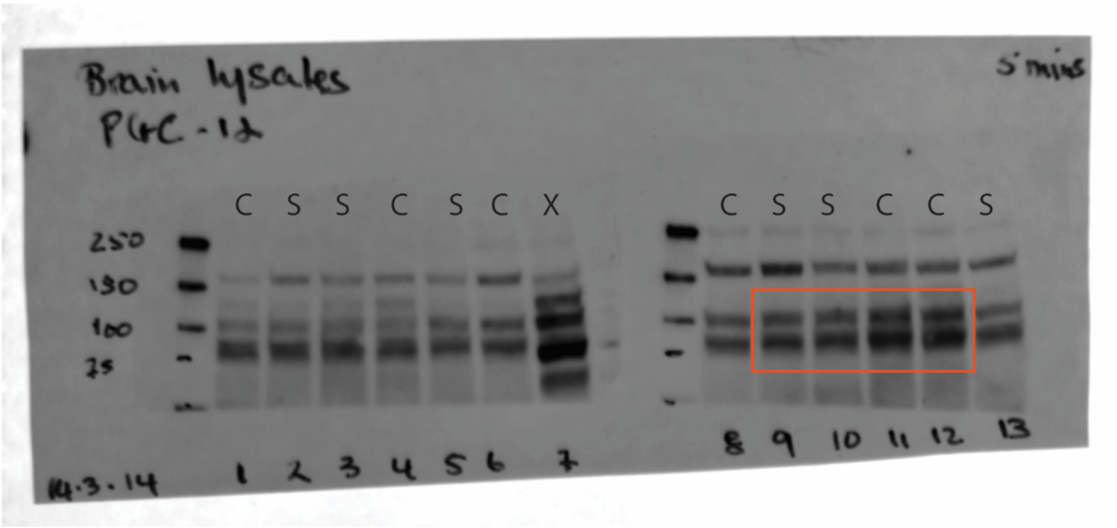

b.

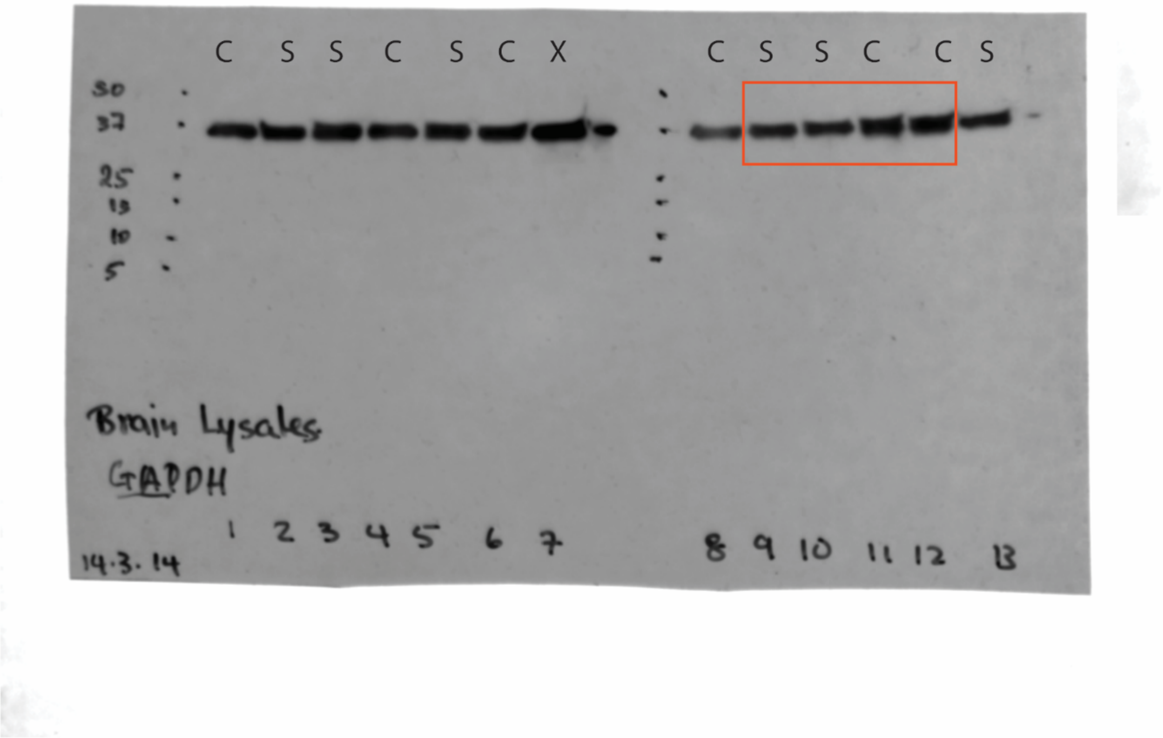

Supplemental Figure 11: Full length membranes used in Western blot analysis of PGC1α. Membranes probed with (**a**) PGC1α and (**b**) GAPDH as a loading control. Red box denotes representative bands used in the main figure image. Labels on each lane indicate sevoflurane exposed (**S**) and control (**C**) samples. All samples were derived from same experiment, with gels/blots processed in parallel. Blinding during analysis is illustrated by the altered ordering of control/sevoflurane samples. An internal sample control is included (“X” marked).

a.

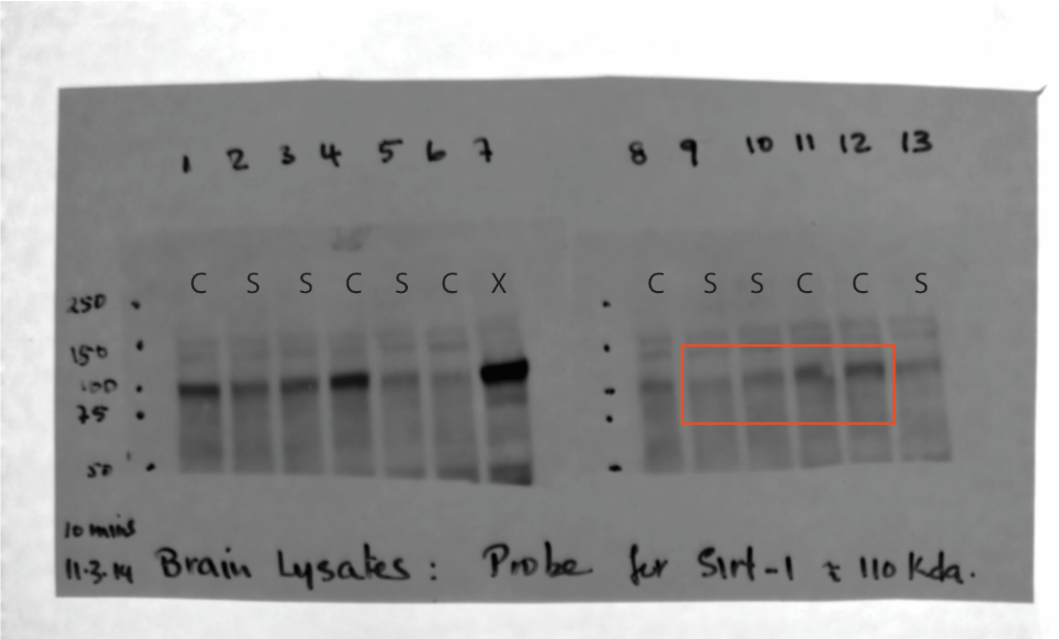

b.

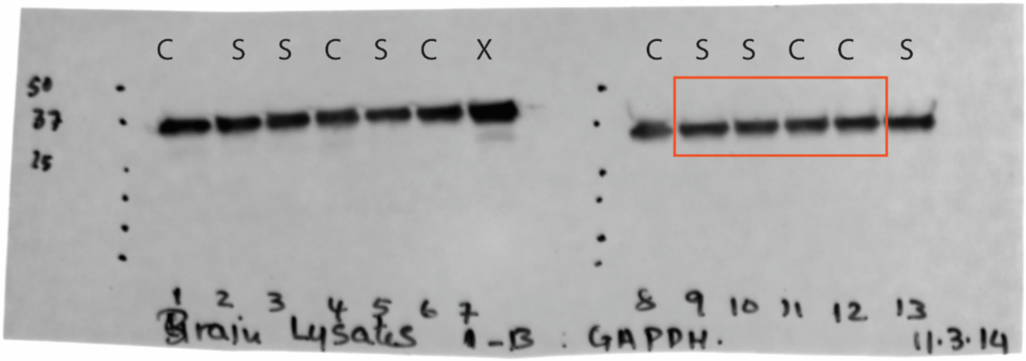

Supplemental Figure 12: Full length membranes used in Western blot analysis of SirT1. Membranes probed with (**a**) SirT1 and (**b**) GAPDH as a loading control. Red box denotes representative bands used in the main figure image. Labels on each lane indicate sevoflurane exposed (**S**) and control (**C**) samples. All samples were derived from same experiment, with gels/blots processed in parallel. Blinding during analysis is illustrated by the altered ordering of control/sevoflurane samples. An internal sample control is included ("X" marked).

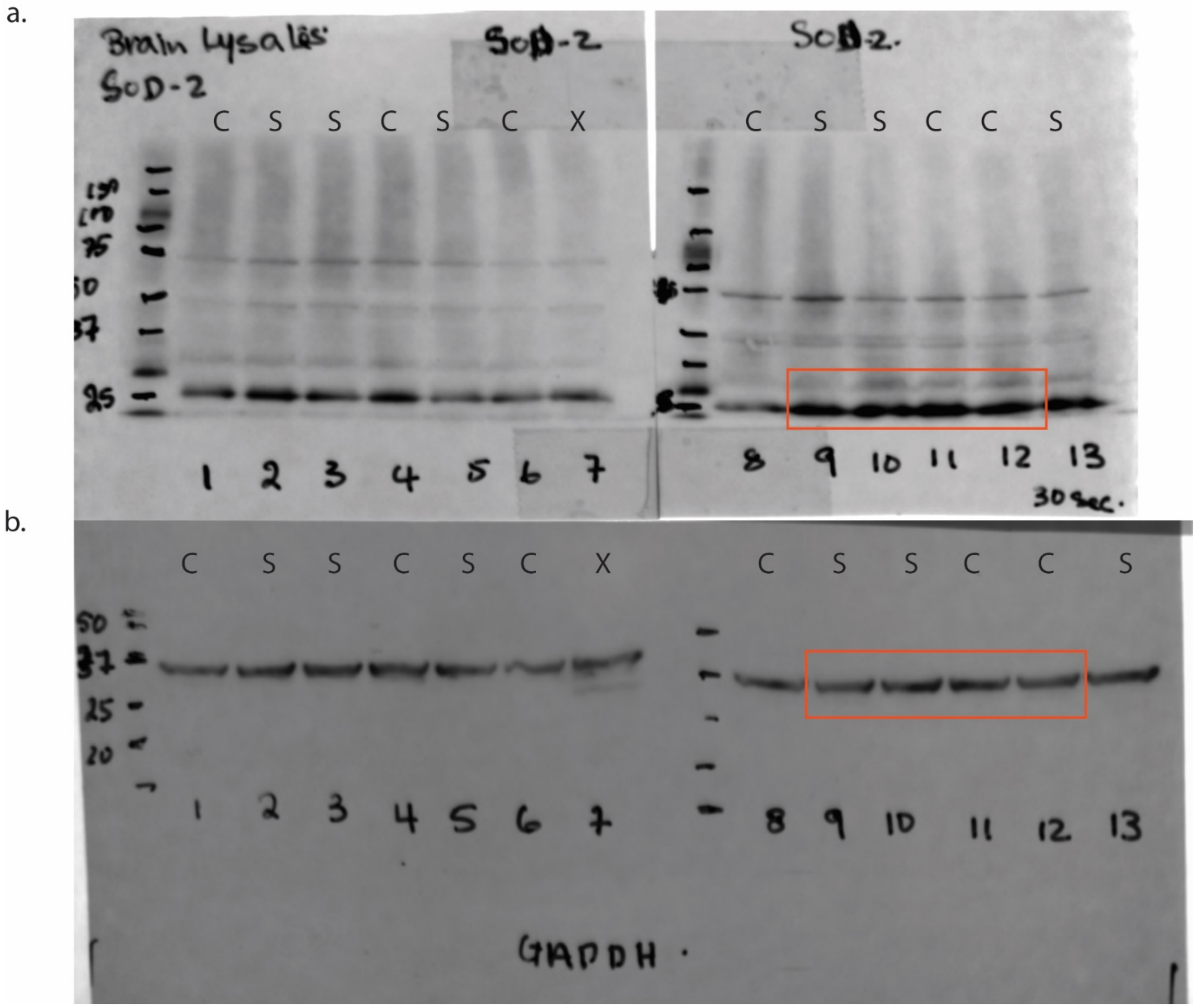

Supplemental Figure 13: Full length membranes used in Western blot analysis of SOD2. Membranes probed with (**a**) SOD2 and (**b**) GAPDH as a loading control. Red box denotes representative bands used in the main figure image. Labels on each lane indicate sevoflurane exposed (**S**) and control (**C**) samples. All samples were derived from same experiment, with gels/blots processed in parallel. Blinding during analysis is illustrated by the altered ordering of control/sevoflurane samples. An internal sample control is included ("X" marked).

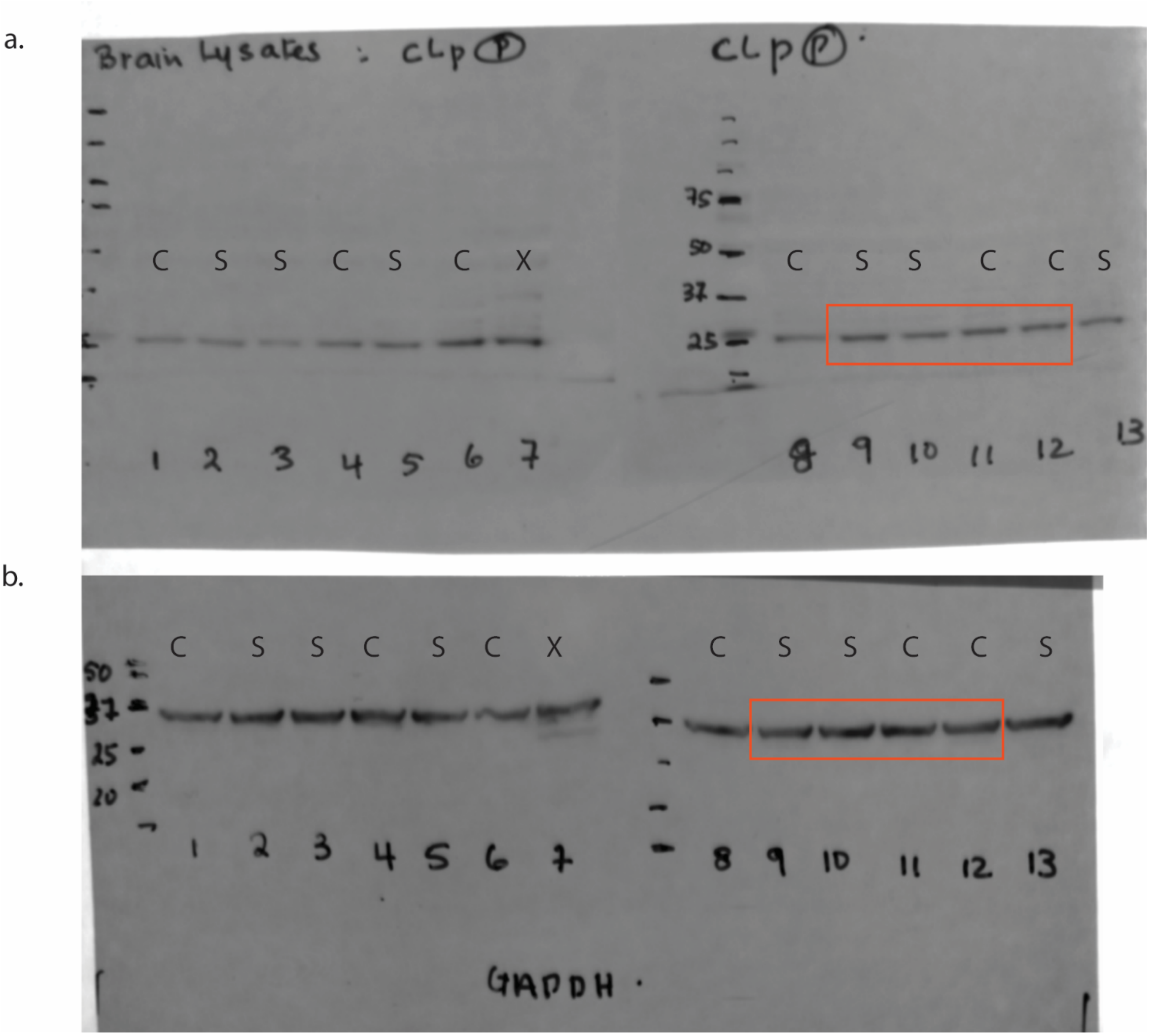

Supplemental Figure 14: Full length membranes used in Western blot analysis of ClpP. Membranes probed with (**a**) ClpP and (**b**) GAPDH as a loading control. Red box denotes representative bands used in the main figure image. Labels on each lane indicate sevoflurane exposed (**S**) and control (**C**) samples. All samples were derived from same experiment, with gels/blots processed in parallel. Blinding during analysis is illustrated by the altered ordering of control/sevoflurane samples. An internal sample control is included ("X" marked).

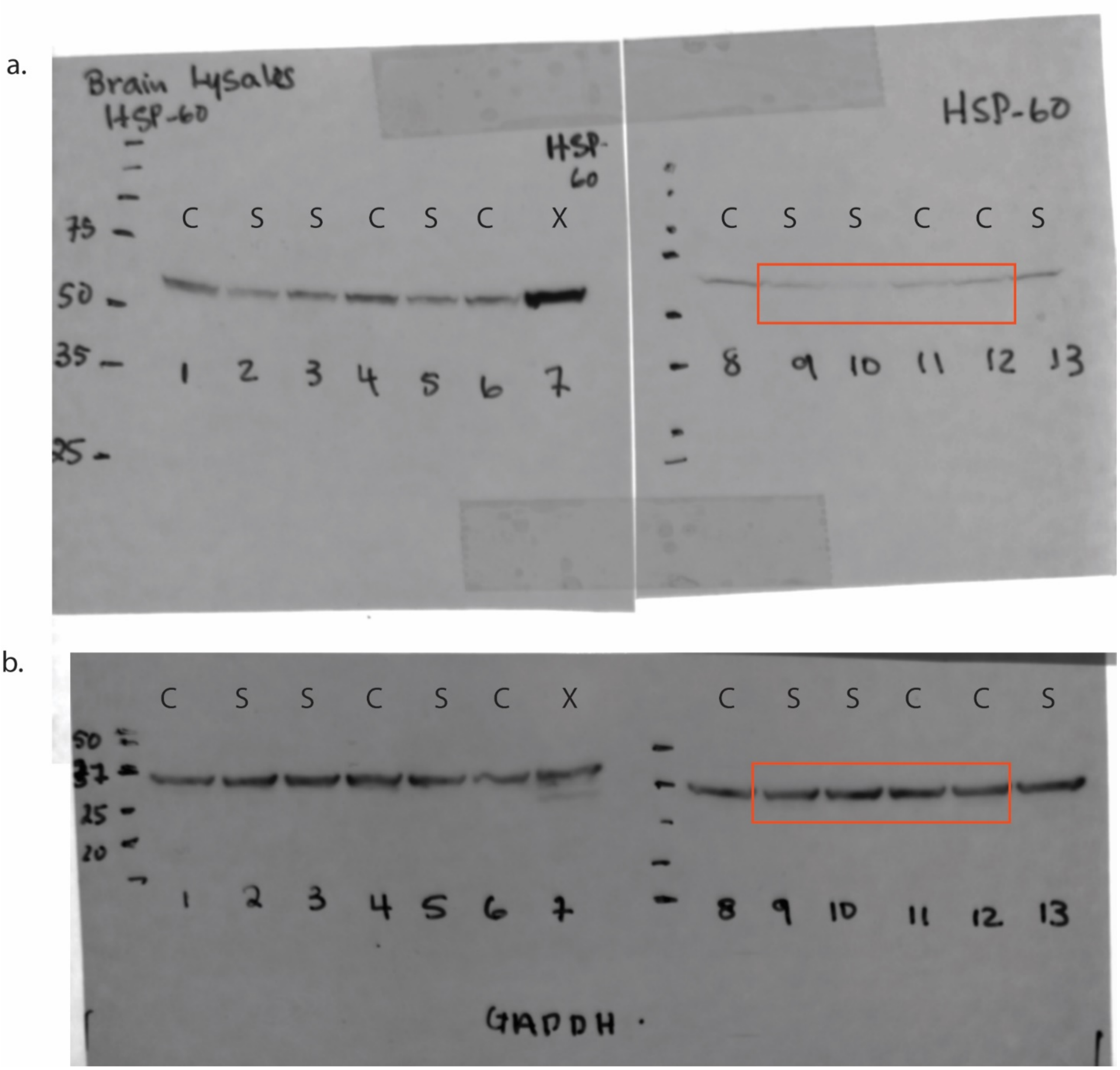

Supplemental Figure 15: Full length membranes used in Western blot analysis of HSP60. Membranes probed with (**a**) HSP60 and (**b**) GAPDH as a loading control. Red box denotes representative bands used in the main figure image. Labels on each lane indicate sevoflurane exposed (**S**) and control (**C**) samples. All samples were derived from same experiment, with gels/blots processed in parallel. Blinding during analysis is illustrated by the altered ordering of control/sevoflurane samples. An internal sample control is included (“X” marked).

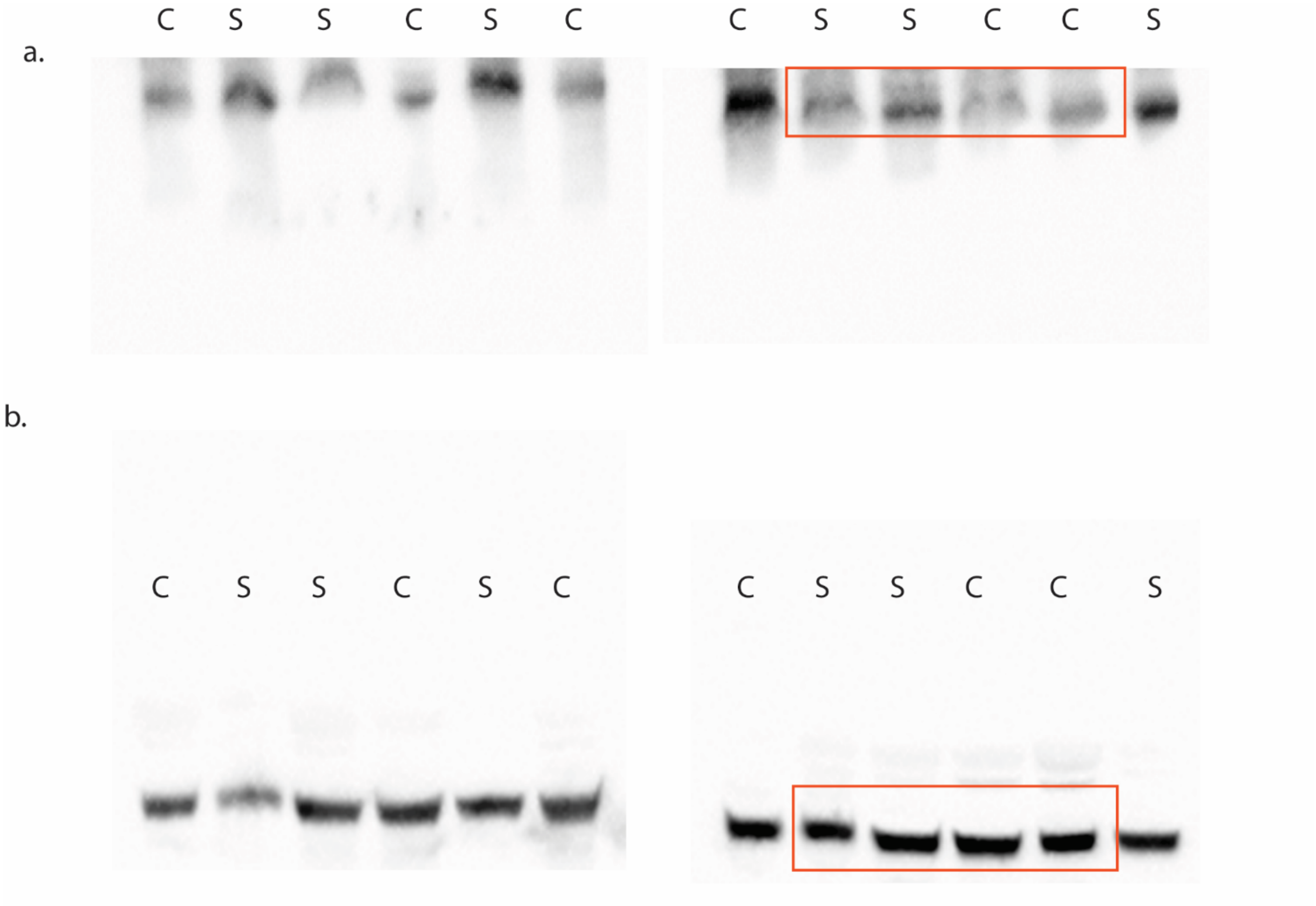

Supplemental Figure 16: Full length membranes used in Western blot analysis of TNFα. Membranes probed with (**a**) TNFα and (**b**) GAPDH as a loading control. Red box denotes representative bands used in the main figure image. Labels on each lane indicate sevoflurane exposed (**S**) and control (**C**) samples. All samples were derived from same experiment, with gels/blots processed in parallel. Blinding during analysis is illustrated by the altered ordering of control/sevoflurane samples.

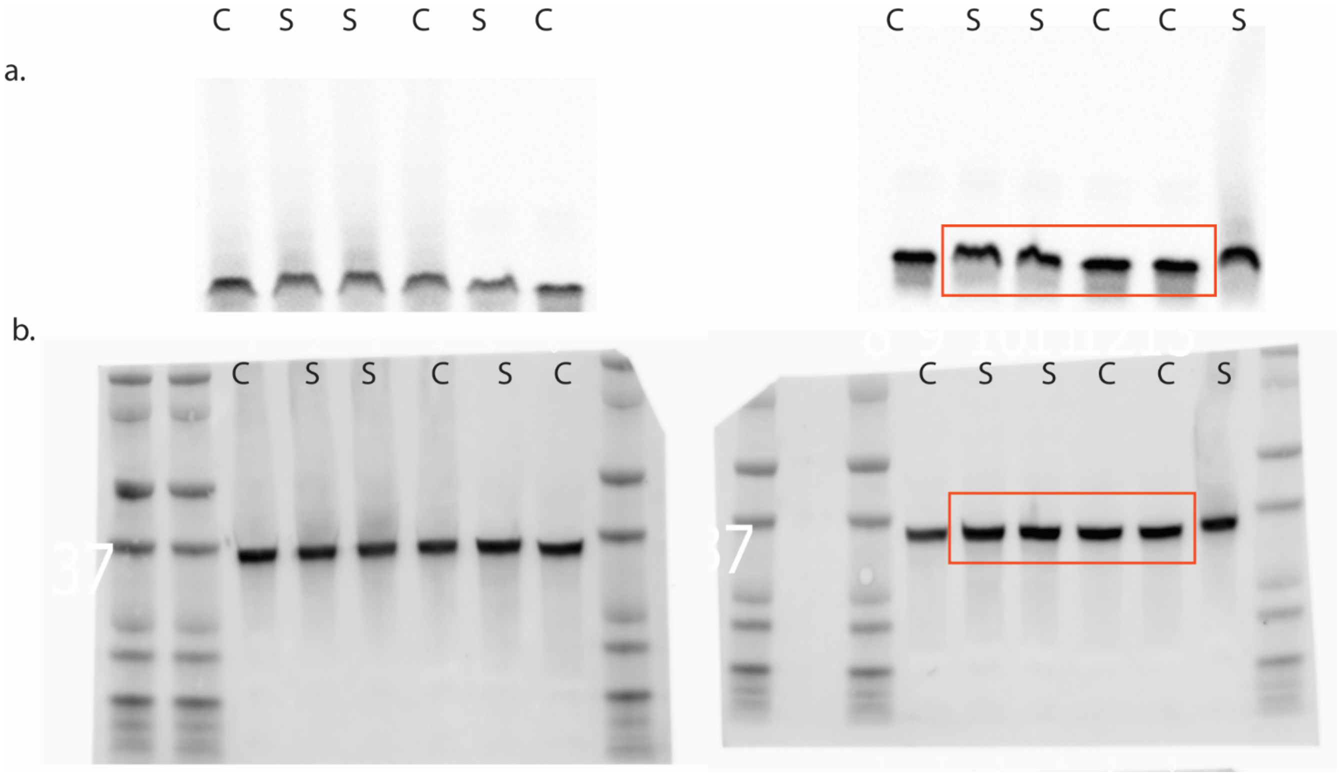

Supplemental Figure 17: Full length membranes used in Western blot analysis of LC3. Membranes probed with (**a**) LC3 and (**b**) GAPDH as a loading control. Red box denotes representative bands used in the main figure image. Labels on each lane indicate sevoflurane exposed (**S**) and control (**C**) samples. All samples were derived from same experiment, with gels/blots processed in parallel. Blinding during analysis is illustrated by the altered ordering of control/sevoflurane samples.

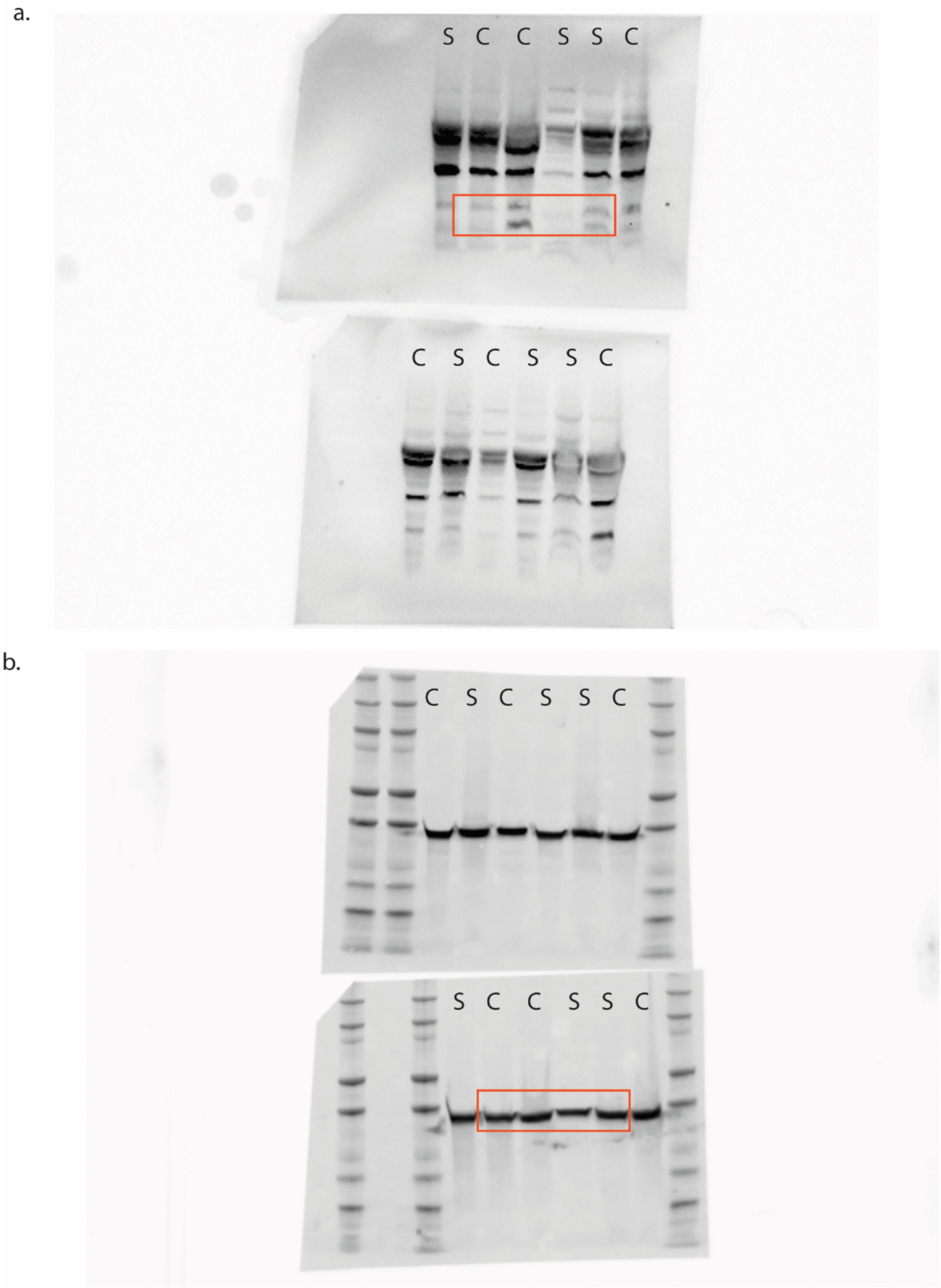

Supplemental Figure 18: Full length membranes used in Western blot analysis of c-Cas3.
Membranes probed with (**a**) c-Cas3 and (**b**) GAPDH as a loading control. Red box denotes representative bands used in the main figure image. Labels on each lane indicate sevoflurane exposed (**S**) and control (**C**) samples. All samples were derived from same experiment, with gels/blots processed in parallel. Blinding during analysis is illustrated by the altered ordering of control/sevoflurane samples.

a.

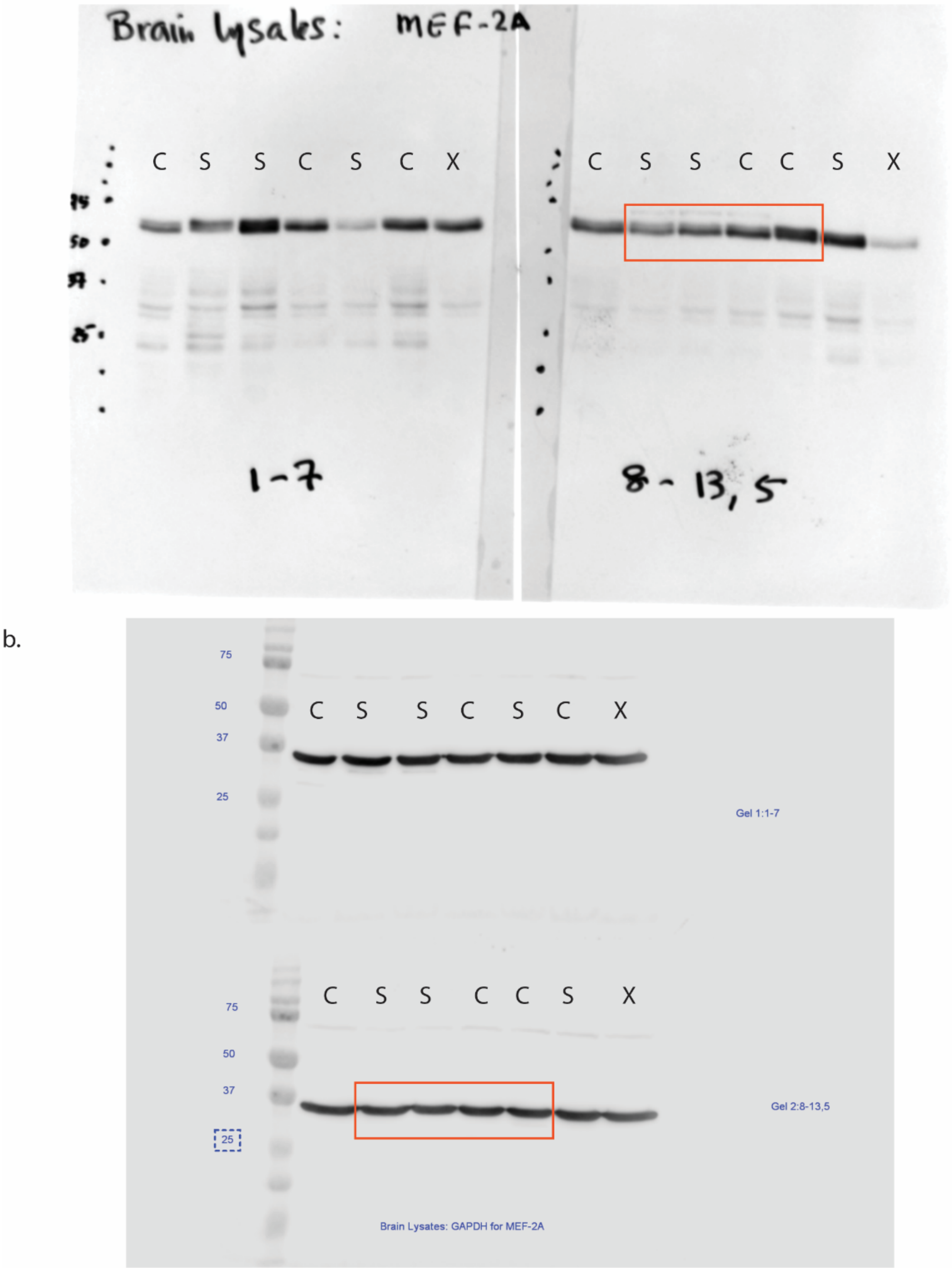

Supplemental Figure 19: Full length membranes used in Western blot analysis of MEF2A. Membranes probed with (**a**) MEF2A and (**b**) GAPDH as a loading control. Red box denotes representative bands used in the main figure image. Labels on each lane indicate sevoflurane exposed (**S**) and control (**C**) samples. All samples were derived from same experiment, with gels/blots processed in parallel. Blinding during analysis is illustrated by the altered ordering of control/sevoflurane samples. An internal sample control is included ("X" marked).

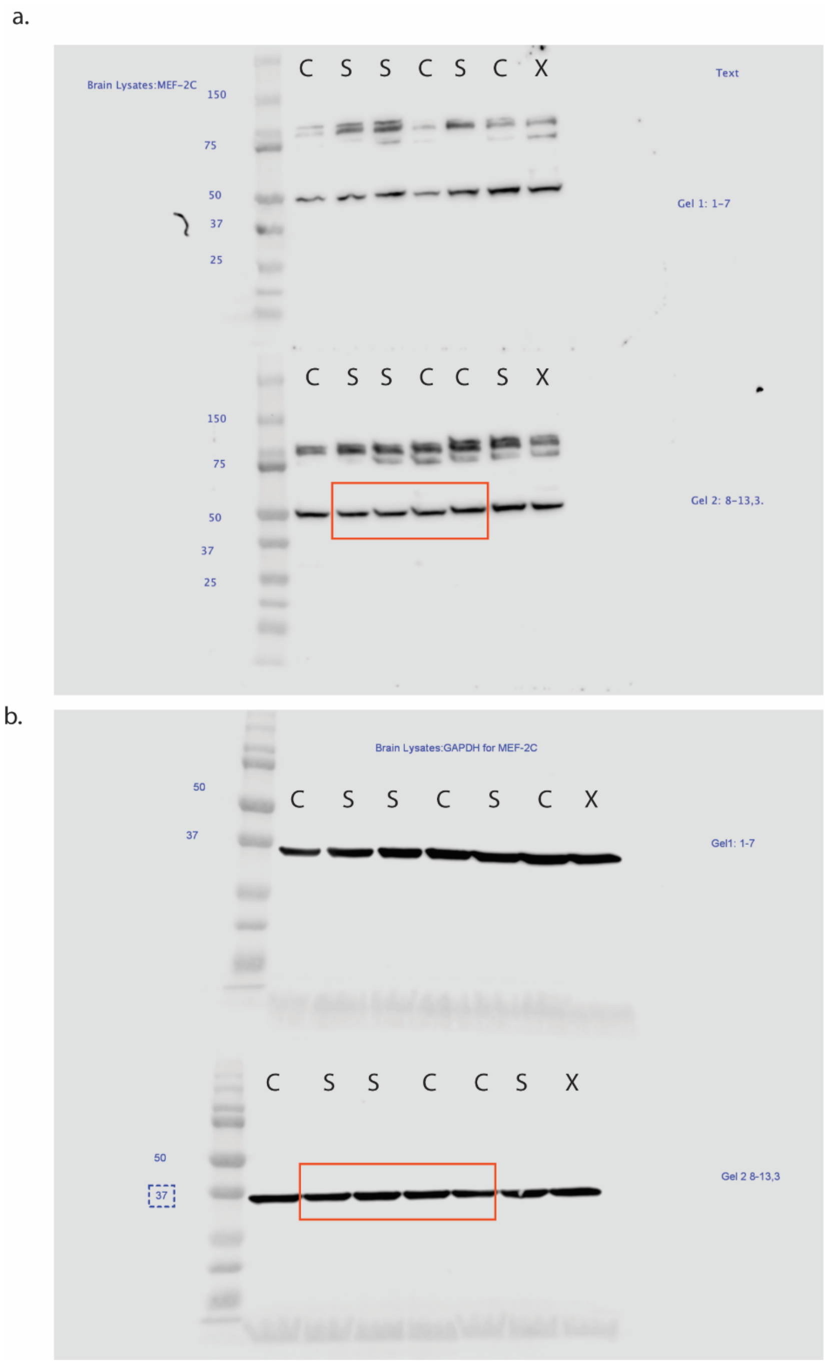

Supplemental Figure 20: Full length membranes used in Western blot analysis of MEF2C. Membranes probed with (**a**) MEF2C and (**b**) GAPDH as a loading control. Red box denotes representative bands used in the main figure image. Labels on each lane indicate sevoflurane exposed (**S**) and control (**C**) samples. All samples were derived from same experiment, with gels/blots processed in parallel. Blinding during analysis is illustrated by the altered ordering of control/sevoflurane samples. An internal sample control is included (“X” marked).

a.

b.

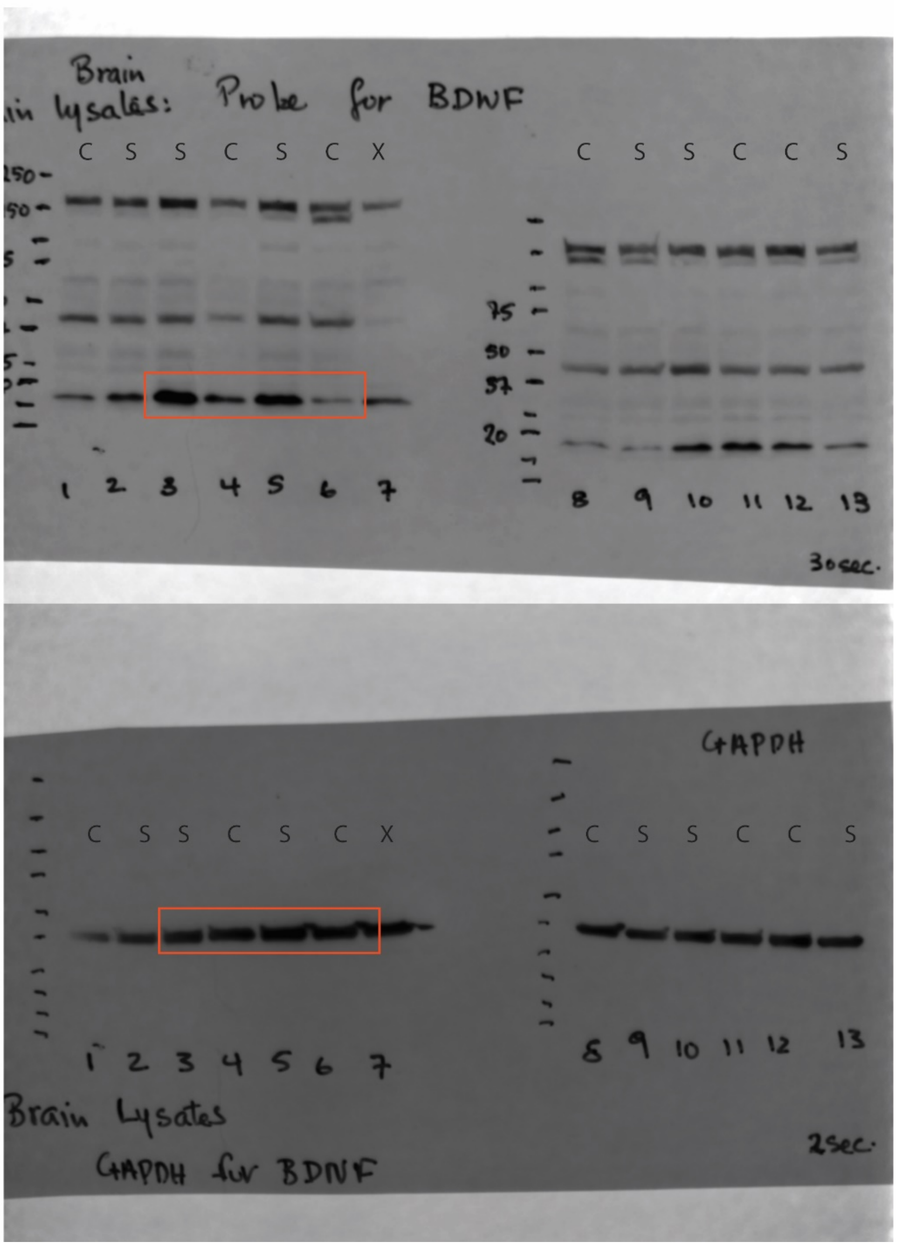

Supplemental Figure 21: Full length membranes used in Western blot analysis of c-BDNF. Membranes probed with (**a**) c-BDNF and (**b**) GAPDH as a loading control. Red box denotes representative bands used in the main figure image. Labels on each lane indicate sevoflurane exposed (**S**) and control (**C**) samples. All samples were derived from same experiment, with gels/blots processed in parallel. Blinding during analysis is illustrated by the altered ordering of control/sevoflurane samples. An internal sample control is included ("X" marked).

Supplemental Table 1. Primary Antibodies Utilized

Antibody	Company	ID	Dilution
NAD-Dependent Deacetylase Sirtuin-1 (SIRT1)	Abcam	Ab139026	1:1000
Peroxisome Proliferator-Activated Receptor Gamma Coactivator 1-Alpha (PGC1α)	Abcam	Ab54481	1:500
Dynamin-Related Protein-1 (DRP-1)	Pierce	PA1-16987	1:500
Optic Atrophy-1 (OPA-1)	Abcam	Ab119685	1:500
Mitochondrial Fission Factor (MFF)	Abcam	Ab139026	1:400
Mitofusion-2 (MFN-2)	Abcam	Ab56889	1:100
Succinate Dehydrogenase Complex A (SDHA)	Santa Cruz	Sc-98253	1:500
Peroxisome Proliferator-Activated Receptor Gamma (PPARγ)	Abcam	Ab24509	1:500
Superdioxide Dimutase-2 (SOD2)	Santa Cruz	Sc-30080	1:500
Clp Protease (ClpP)	Santa Cruz	Sc-134496	1:200
Heat Shock Protein 60 (HSP60)	Santa Cruz	Sc-13115	1:200
Brain-Derived Neurotrophic Factor (BDNF)	Abcam	Ab-46176	1:1000
Myocyte Enhancer Factor-2A (MEF2-A)	Abcam	Ab-109420	1:300
Myocyte Enhancer Factor-2C (MEF2-C)	Abcam	Ab-64644	1:100
Estrogen Receptor Alpha (EERα)	Abcam	Ab-76228	1:500
Tumor Necrosis Factor Alpha (TNFα)	Santa Cruz	Sc-52746	1:100
Caspase 3 (Cas3)	Abcam	Ab-13847	1:500
Phosphorylated Dynamin-Related Protein-1 (pSer616-DRP1)	Cell Signaling	#3455s	1:1000
Light Chain 3B (LC3B)	Cell Signaling	#2775	1:1000
Glyceraldehyde 3-Phosphate Dehydrogenase (GAPDH)	Sigma	G8795-25UL	1:5000

Supplemental Table 2. Primers designed to amplify rat mitochondrial targets

Primer	Sequence (5'- 3')
ND1 Forward	ATGCCATAAAACTATTCATAAAAGAAC
ND1 Reverse	ATTCATATGGCTTAGAGCTAGTGT
GAPDH Forward	ATGACTCTACCCACGGCAAG
GAPDH Reverse	GGAAGATGGTGATGGGTTTC
